# Voluntary running wheel exercise induces cognitive improvement post traumatic brain injury in mouse model through redressing aberrant excitation regulated by voltage-gated sodium channels 1.1, 1.3, and 1.6

**DOI:** 10.1007/s00221-023-06734-2

**Published:** 2023-11-23

**Authors:** Dan Wang, Hui-Xiang Zhang, Guo-Ji Yan, Hao-Ran Zhao, Xiao-Han Dong, Ya-Xin Tan, Shan Li, Min-Nan Lu, Rong Mei, Li-Na Liu, Xu-Yang Wang, Yan-Bin Xiyang

**Affiliations:** 1 Institute of Neuroscience, Faculty of Basic Medical Science, Kunming Medical University, 1168 West Chunrong Road, Yuhua Avenue, Chenggong, Kunming, Yunnan 650500 People’s Republic of China; 2 Department of Anatomy, Changsha Medical University, Changsha, China; 3 Department of Pediatrics, The People’s Liberation Army (PLA) Rocket Force Characteristic Medical Center, Beijing, 100088 China; 4 Science and Technology Achievement Incubation Center, Kunming Medical University, Kunming, Yunnan 650500 China; 5 Department of Neurology, The First People’s Hospital of Yunnan Province, Kunming, Yunnan China; 6 Department of Neurosurgery, Shanghai Sixth People’ Hospital Affiliated to Shanghai Jiao Tong University School of Medicine, Shanghai, 200233 People’s Republic of China

**Keywords:** Traumatic brain injury, Sodium current, Neuronal excitability, Voltage-gated sodium channels, Cognitive function

## Abstract

Traumatic brain injury (TBI) leads to disturbed brain discharge rhythm, elevated excitability, anxiety-like behaviors, and decreased learning and memory capabilities. Cognitive dysfunctions severely affect the quality of life and prognosis of TBI patients, requiring effective rehabilitation treatment. Evidence indicates that moderate exercise after brain injury decreases TBI-induced cognitive decline. However, the underlying mechanism remains unelucidated. Our results demonstrate that TBI causes cognitive impairment behavior abnormalities and overexpression of Nav1.1, Nav1.3 and Nav1.6 proteins inside the hippocampus of mice models. Three weeks of voluntary running wheel (RW) exercise treatments before or/and post-injury effectively redressed the aberrant changes caused by TBI. Additionally, a 10% exercise-conditioned medium helped recover cell viability, neuronal sodium current and expressions of Nav1.1, Nav1.3 and Nav1.6 proteins across cultured neurons after injury. Therefore, the results validate the neuroprotection induced by voluntary RW exercise treatment before or/and post-TBI. The RW exercise-induced improvement in cognitive behaviors and neuronal excitability could be associated with correcting the Nav1.1, Nav1.3, and Nav1.6 expression levels. The current study proves that voluntary exercise is an effective treatment strategy against TBI. The study also highlights novel potential targets for rehabilitating TBI, including the Navs proteins.

**Supplementary Information:**

The online version contains supplementary material available at 10.1007/s00221-023-06734-2.

## Introduction

Traumatic brain injury (TBI) involves the functional disruption or pathological changes in the brain due to an external physical force. This could involve road traffic incidents, accidental falls, and other injuries. Therefore, TBI is a significant global health problem, becoming and a leading cause of death and disability in trauma patients (Gao et al. 2020; Khellaf et al. 2019). TBI can be characterized as mild, moderate, and severe depending on injury severity (Lee et al. 2019). Unfortunately, effective treatments to combat TBI-related disorders are still lacking (Chytrova et al. 2008).

Since physical rehabilitation is one of the only established therapies for TBI, its efficacy requires better optimization. Exercise is beneficial to overall health and has been associated with cognitive function. Voluntary wheel running in mice is intermittent and resembles interval training among humans (Manzanares et al. 2018). Effective pharmacotherapies can be facilitated through a more comprehensive understanding of mechanisms behind exercise-related cognitive improvement due to exercise post-TBI.

Voltage-gated sodium channels (VGSCs) transmembrane protein complexes, including Nav1.1–1.9 plus Nax subtypes, propagate action potentials in excitable cells (Manzanares, Brito-da-Silva and Gandra 2020). VGSCs are highly activated by TBI and are associated with TBI-induced pathological progress. For instance, Nav 1.3 and 1.6 messenger ribonucleic acid (mRNA) and protein expression were considerably up-regulated in the ipsilateral-injured cortex of rats during the very early stage post-TBI. Thus, it is linked with TBI severity and associated with diffuse axonal injury post event in mice (Huang et al. 2013; Mao et al. 2010a, b; Wolf et al. 2001). Mechanical trauma of axons leads to a Na + influx through VGSCs and subsequently triggers Ca^2+^ influx, causing neuronal death (Iwata et al. 2004). Our previous study observed that exercise-induced cognitive improvement is related to sodium channel-mediated excitability (Tan 2020). However, the correlation between voluntary RW exercise-induced cognitive recovery in the TBI model remains unknown. Therefore, whether voluntary RW exercise recovered cognitive function after TBI by correcting the VGSCs is unconfirmed.

In the current study, mice were treated with voluntary RW before or after TBI or combined before and after injury. Subsequently, the behavioral changes, electroencephalography (EEG) electrophysiology recording, and VGSC expression levels in mice were evaluated. Primary cultured neurons helped establish an in vitro TBI model. Neuronal hyperexcitability, sodium current, and protein expression of VGSCs in TBI-injured neurons helped determine the impact of serum cell viability in exercising mice.

## Materials and methods

### Animal grouping for in vivo experiments

A total of 252 C57BL/6 J male mice aged 4–6 weeks, were randomly divided into exercise pre-training, post-injury exercise intervention, and pre-training combined with TBI post-injury exercise training groups. The PASS software helped determine the sample size. The calculation details and the number of mice in each group are demonstrated in Supplementary Tables 1, 2, 3. Each group involved sham-operated and TBI mice. Mice in the sham-operated group only received craniotomy without TBI injury. However, TBI mice suffered from a mild TBI operation. Moreover, the experimenters did not inform the treatment of experimental animal groupings.

All the cages had a runner wheel (RW) (diameter = 12 cm, width = 5 cm; Nalge Nunc International, Rochester, NY, USA). It was used for voluntary exercise and free rotation. As previously reported, it was attached to a receiver to monitor the number of revolutions (Vital Viewer Data Acquisition System software, Mini Mitter, Sunriver, OR, USA) (Bao et al. 2014; Hu et al. 2015). The mice exercised ad libitum in individual cages with unlimited RW access. The mean revolutions were determined from 7 p.m. to 7 a.m. for the most active period every night. Mice from the sedentary (SED) or Non-runners (NR) control group were exposed to fixed and non-rotating RW.

In the exercise pre-training group, the mice were housed with voluntary access to an RW or an immobilized RW for three weeks prior to the operation. In the post-injury exercise intervention group, the mice were housed with voluntary access to an RW (Runner, R) or an immobilized RW (Non-Runner, NR) for 3 weeks post-injury. In the mixture exercise training group, mice were treated with voluntary RW exercise before and after TBI for 3 weeks each (PreS + Non-Runner, PS NR; Pre + Runner, PR). The mice in the SED control group were exposed to fixed and non-rotating RW.

The mice treated with preliminary voluntary RW exercise were anesthetized with isoflurane inhalation and decapitated at 2, 6, 12 or 24 h post-operation (hpo). The ipsilateral hippocampus in the exercise pre-training group was removed and prepared to depict the Nav1.1, 1.3, and 1.6 protein expression levels (*n* = 9).The mice were prepared for recording (EEG) or behavioral tests (open field, Morris water maze, and object recognition test) at 21 days post-operation (dpo). After behavioral evaluation, the mice were decapitated, and the ipsilateral hippocampus was removed to undergo Western blotting. Besides, excitatory postsynaptic potentials (fEPSPs) (long-term potential) were recorded in the CA1 of the hippocampal slice at 24 hpo and 21 dpo.

Behavioral tests (open field, Morris water maze and novel object recognition test), EEG and fEPSPs recording in the CA1 of the hippocampal slice were performed at 21 dpo in mice treated with voluntary RW exercise for 3 weeks post-TBI. Then, the protein expression levels of Nav1.1, 1.3, and 1.6 in the ipsilateral frontal cortex and hippocampus were assessed post-behavioral evaluation.

Moreover, behavioral tests, EEG tests, and fEPSPs recording in the CA1 of the hippocampal slice were undertaken in mice treated with voluntary RW exercise before and after TBI for 3 weeks each. Then, the mice were sacrificed to determine the Nav1.1, 1.3, and 1.6 protein expression levels in the ipsilateral frontal cortex and hippocampus. The experimenters or data processors were blinded to the animal grouping information to ensure unbiased results.

### Animal grouping for in vitro experiments

We randomly chose 18 C57BL/6 J male mice aged 4–6 weeks as serum donors for cell culture. These mice were uniformly divided into free exercise pre-training (Runners, R) and sedentary control groups (Non-runners, NR) groups. Exercising mice possessed voluntary access to the RW, while the SED control ones were accessible to the same RW in the locked position. The mice could exercise ad libitum in the individual cages for three weeks with unlimited access to the RW. After training, the mice from each group were sacrificed to obtain exercise-conditioned (Runners, R) or non-exercise-conditioned (Non-runners, NR) serum.

### TBI operation

The mild-TBI mouse model was performed following our previous study (Hu et al. 2015). Surgical anesthesia was started by exposing mice to a mixture of 3% isoflurane in oxygen within an incubation chamber. Anesthesia was maintained with 1–2% isoflurane in oxygen during surgery using an isoflurane vaporizer (Riward R580S, Shenzhen, China). After anesthesia, the animals were fixed on a stereotactic platform (Riward 71,000, Shenzhen, China). Then, the skull was opened to expose the corticomotor area in the cerebral cortex. A mild-TBI mouse model was developed with a device to generate controlled cortical impact (CCI), allowing independent tissue deformation and impact velocity manipulation. Then, the left parietotemporal cortex was subjected to CCI [2 mm tissue deformation at a rate of 6.0 m/s (moderate)]. It possessed a depth of 1 mm and a contact time of 200 ms or Sham surgery (Fox et al. 1998; Hu et al. 2015). After the operation, the incision was closed with interrupted 6–0 silk sutures. The anesthesia was terminated, and the animal was placed inside a heated cage to maintain normal core temperature for 45 min post-operation. All the animals were carefully monitored everyday post awakening. The same modeler underwent preoperative anesthesia and CCI modeling over a short time (3 days) to decrease experimental practice differences.

## Behavioral tests

### Open field assessment

As previously described, the open-field assessment tested anxiety-like behavior and locomotor activity (Hazra et al. 2014). The mice were placed inside a brightly lit circular arena (40 × 40 × 40 cm^3^) to evaluate their general activity levels while assessing their gross locomotor and exploratory activity in each group. The horizontal (entire distance traveled) and vertical (the number of rearing up movements) activities were deciphered and quantified using the TOPSCAN tracking system (Clever Sys Inc., USA) over a 10-min trial period. The number of fecal balls was evaluated during the 10-min trial period. The stationary behavior is a transient state during which animals are immobile and do not engage in defined behavior. This behavior was also evaluated (Hazra et al. 2014).

### Morris water maze (MWM) test

The MWM possesses a circular pool (100 cm in diameter, 50 cm deep) filled with white water at 24 ± 1 °C at 20 cm depth (XR-XM101-Z; Shanghai XinRuan Information Technology Co., Ltd., China). The MWM test was run as previously demonstrated (XiYang et al. 2016). All the mice underwent a preliminary experiment for water adaptation. This helped them to step on a white platform (hidden below the water surface) and escape a day before the regular testing. For this investigation, a mouse was allowed to swim in the water for 10 s. Then, they were placed on a white platform submerged under water for 1 to 2 s. The hidden platform was placed in a different location for regular testing. The time during which the mice discovered the submerged white platform was recorded as escape latency, recorded for 5 days. On testing day 6, the platform was removed for the probe trial. Each mouse was allowed to swim for 60 s to evaluate its platform location memory. Percentages of time spent in the target quadrant, path, number of target platform crossings, and the swim speed (mm/s) were documented. The time spent and distances traveled in the four quadrants were also noted. The animals were tracked using an overhead video camera, and an Animal behavior video analysis system helped analyze the data (Shanghai XinRuan Information Technology Co., Ltd., China).

### Novel object recognition (NOR) task

As previously described, NOR tasks were performed within the same arena (40 × 40 × 40 cm^3^) for the open-field assessment (Figueiredo et al. 2013; Lourenco et al. 2013). Test objects (4 or 5 cm in height) were glass or plastic with different shapes, textures, colors and sizes. The mice were placed at the arena center with two identical objects during the initial training period of 5 min. Trained researchers independently determined the time required to explore each object. At 1 h post-training, the mice were reinserted into the arena for a 10 min test session. One of the objects was replaced by a novel one in this session. It had a different shape and color but was consistent in height. Object exploration was characterized as the direct contact of the nose or front paws with the object. During the formal testing phase, the exploration time of mice toward novel or familiar objects was measured to determine the Discrimination Index (DI). The specific calculation formula is DI = (NT–FT) / (NT + FT), where NT indicates the time spent by mice exploring the new object and FT indicates the time spent exploring the familiar object. The obtained DI helps in comprehending understand the cognitive ability of mice.

## Electrophysiological recordings for mice

### EEG recordings

Mice underwent EEG implantation surgery seven days prior to their final time point, 14 dpo. A three-channel two twisted bipolar stainless-steel electrode used with a diameter of 0.010 in (P Technologies) was used in all the EEG implantations. The protocols of electrode implantation and EEG recordings protocols followed the literature with certain modifications (Hu et al. 2017; Lee et al. 2011; Szu et al. 2020a, b; Tan et al. 2020). The 2-mm twisted bipolar wires were implanted in the ipsilateral dorsal hippocampus. The 0.5-mm untwisted wire was utilized to ground within the cortex. Approximately, 0.5 mm of the insulating coat was removed from the distal wire tip to achieve reliable EEG recordings (Szu et al. 2020a, b). Before EEG recordings, the mouse was fixed on the stereotaxic instrument for electrode fixation after being anesthetized. First, the middle scalp was incised about 2 cm to expose the skull. The bregma and lambda points (electrode position on the surface of the mouse brain) were identified on the skull based on the mouse brain atlas (Paxinos and Franklin’s The Mouse Brain in Stereotaxic Coordinates). Then, the mice were implanted with an electrode pedestal into the hippocampus (AP-2.1 mm, ML-1.5, DV-1.5 mm) until it rested on the skull. Next, two micro screws, used as ground and reference, were fixed on the occipital bone above the cerebellum region.

The mouse with the microelectrode implant recovered for 6 days post operation. The connector was linked with a multi-channel EEG amplifier system to gather, and the neural electrical signals. EEG acquisition began after recovery, and the signal was collected in freely moving mice lasting 24 h.

The original recording signal obtained by the recording electrode is amplified 5000 × with the amplifier. The analog signals were digitized at a sampling frequency rate of 1 kHz using a CED Micro1401 data acquisition system and an A/D sampling rate of 128 Hz. Moreover, the signal became a band-passed filter between 0.1 and 35 Hz, digitized at 625 samples/s.

The power spectral density (PSD) analyses were performed blind. The resting EEG data acquired during the 24 h was extracted using the AcqKnowledge software (Biopac), and PSD was analyzed with BrainVision Analyzer 2.1 (Brain Products). The artifacts were removed using a semiautomatic procedure based on maximum-minimum, low activity, and amplitude criteria. The EEG signals were divided into 10-s segments, and a fast Fourier transform was applied to each segment at 1-Hz resolution from 1 to 35 Hz to generate the frequency values. The EEG frequency bands were delta (1–3 Hz), theta (4–7 Hz), alpha (8–14 Hz), and beta (15–35 Hz). The fast Fourier transform results were extracted using the BrainVision Analyzer 2.1 and averaged using specialized MATLAB scripts (MathWorks).

### Hippocampal field excitatory postsynaptic potential (fEPSP) recording

The synaptic functions were systematically analyzed in the hippocampus to identify whether hippocampal synaptic plasticity was affected by voluntary RW exercise in TBI mice (XiYang et al. 2016). The fEPSP was recorded at CA1 Schaeffer collateral synapses within hippocampal slices collected from mice at 24 h and 21 d after undergoing the operation. Hippocampal slices (350 μm) were cut using a vibrating microtome in an ice-cold slicing buffer bubbled with 95% O_2_ and 5% CO_2_. Then, the slices were transferred to a holding chamber possessing oxygenated artificial cerebrospinal fluid for 45 min at 34 °C and another 45 min at 22 °C for recovery. Later, it was transferred to a submersion recording chamber while continually perfusing with 32 °C oxygenated ACSF. The slices were equilibrated before each recording for at least 15 min. ACSF-filled glass electrodes (resistance < 1 MΩ) were positioned inside the stratum radiatum of the CA1 area for extracellular recording. The synaptic responses were evoked by stimulating the Schaeffer collateral with 0.2 ms pulses using a bipolar tungsten electrode (WPI Inc., Sarasota, FL) once every 15 s. The stimulation intensity was systematically enhanced to identify the maximal fEPSP slope and adjusted to provide 50% of the maximal fEPSP slope. The experiments with maximal fEPSPs < 0.5 mVor using substantial changes in the fiber volley were rejected. Long-term potential (LTP) was provoked using one 1 s/100 Hz stimulus train for 60 min after a stable baseline recording for 20 min.

### Primary neurons culture in *vitro*

The cortical or hippocampal tissues were retrieved from postnatal day 0 (P0) C57BL/6 J mice (Kim and Magrané, 2011). The crushed cortical and hippocampal tissues were digested using 1.25% pancreatin (Gibco, ThermoFisher Scientific) inside an incubator at 37 °C for 10 min before being resuspended with fetal bovine serum (Gibco). The resulting tissue suspension was filtered using a 70 μm cell strainer to gather the cortical or hippocampal cells. These cells were incubated inside six-well culture plates pre-coated with poly-D-lysine (Sigma-Aldrich). They were soaked with neuronal medium (Gibco) possessing 2% B27 supplement (Gibco), 0.5% penicillin/streptomycin, and 0.25% GlutaMax (Gibco). Half of the culture medium was replaced every three days. Then, the cultured cells underwent a mechanical injury (trauma) after 10–12 days to mimic TBI in vitro (Katano et al. 1999; Mori et al. 2002). A sterile 21-gauge needle was used to from parallel scratches across circular wells of culture plates (9 × 9 scratches in six-well and 6 × 6 scratches in 12-well plates). The control wells were left uninjured, and the medium was replaced using serum-free DMEM/F12 and incubated at 37 °C.

### Effects of the exercise-conditioned medium on primary cortical cultures

The mice from the Runners or Non-Runners group were sacrificed to obtain exercise- (R) or non-exercise-conditioned (NRs) serum (*n* = 9) (Hojman et al. 2011). Blood was collected by decapitation, followed by quick excision, dry ice freezing and sorting in 100% alcohol. The serum from the exercising mice was pooled in the groups. It was followed by heat inactivation at 56 °C for 30 min and sterile filtration. At 2, 6 or 24 h after mechanical injury, the primarily injured cortical neurons were treated with using 1 ml medium having 10% exercise-conditioned or non-exercise- serum instead of the neurobasal medium (Chen et al. 2017; Hojman et al. 2011). The cells were implanted in 12-well plates with 1 × 10^5^ cells per well.

Uninjured (intact) neurons were spread across 12-well plates (1 × 10^5^ cells per well), incubated for 24 h, and washed thrice with the prewarmed neurobasal medium. The intact cultures were exposed for 2, 6 or 24 h in 1 ml of a mixed medium containing 10% exercise- or non-exercise-conditioned serum. The control cells were incubated in 1 ml of the fresh neurobasal medium. The cells were incubated for 2, 6 or 24 h within the medium for 3-(4,5-dimethylthiazol-2-yl)-2,5-diphenyltetrazolium bromide (MTT) assay, electrophysiological recording and Western blotting assay.

### 3-(4,5-Dimethylthiazol-2-yl)-2,5-diphenyltetrazolium bromide (MTT) assay

The cell viability was determined using the MTT assay kit (Sigma-Aldrich) based on the manufacturer’s guidelines. The primary neuronal cells were seeded in 96-well plates at 2 × 10^4^ cells per well and treated with various exogenous substances depending on the following protocol. The MTT reagent was added to the cells at 37 °C post-incubation. The culture medium was replaced with 100 μL DMSO to dissolve the formazan crystals after incubating for 4 h. The absorbance of each well was determined at 570 nm using a microplate reader (Microplate reader, Bio-Rad, 3550).

### Electrophysiological recording in vitro

Electrophysiological assessments could detect action potentials or sodium currents in the primary cortex or hippocampal neurons at room temperature. As described previously, the electrophysiological patch clamp recording was performed (Hu et al. 2020; Shan et al. 2019). Briefly, the cells were patched in voltage-clamp mode and regulated at − 70 mV. The membrane capacitance (Cm), input resistance (Rm), and time constant (tau) were measured by applying the depolarization step voltage command (mV) and using the pClamp10 software integration membrane test function. Next, the recordings were switched to the current clamp mode. The resting film potential was adjusted to  – 80 mV by introducing a positive current (50–100 pA). The, a series of depolarizing current pulses were used. The intrinsic excitation was determined by developing the input–output (i–o) function.

### Western blotting

After the EEG recording, the 4–6-week-old C57BL/6 J mice were sacrificed by cervical dislocation and decapitation. The brains were rapidly kept in ice-cold phosphate-buffered saline (PBS) inside a sterile dish for western blotting. The injured or intact neurons treated with or without an exercise-conditioned medium were harvested for western blotting. The manufacturer helped to determine the specificity of the primary antibodies, and previous reports depicted by amino acid and/or mass spectrometry analysis (https://www.alomone.com/p/anti-nav1-1-2/ ASC-001?b = 390) The tissues or cells were homogenized separately on ice in lysis buffer possessing 10% sodium dodecyl sulfate (SDS), 10 mM Tris–HCl buffer (pH 7.4), 30% Triton-1000, 10 mM ethylenediaminetetraacetic acid (EDTA), protease inhibitor cocktail (Roche, Switzerland), and NaCl, using a homogenizer. The homogenates were centrifuged at 5000 g for 10 min at 4 °C. The protein was quantified using the bicinchoninic acid reagent (Sigma-Aldrich; Merck KGaA, Darmstadt, Germany) method. The equal protein amounts were resolved using SDS–polyacrylamide gel electrophoresis (PAGE) on 4–12% gels and transferred to nitrocellulose membranes. Then, they were incubated against Nav1.1 (1:800; cat. no. ASC-001; Alomone), Nav1.3 (1:500; cat. no. ASC-004; Alomone), or Nav1.6 (1:600; cat. no. ASC-009; Alomone) using antibodies. GAPDH (mouse monoclonal anti-GAPDH; 1:800; no. sc-47724, Santa Cruz, Delaware, CA, USA) was used as a reference. The membranes were incubated with matched secondary antibodies for 2 h at 20–25 ˚C. Horseradish peroxidase-conjugated anti-rabbit antibodies used to detect Nav1.1, Nav1.3, and Nav1.6 (1:2,500; cat. no. PI-1000; Vector Laboratories, Inc.). A peroxidase-conjugated anti-mouse secondary antibody (1:3,000; cat. no. PI-2000; Vector Laboratories, Inc.) was used for β-actin detection. Elevated chemiluminescence luminol reagent (Beyotime Institute of Biotechnology, Shanghai, China) helped quantify protein. A Bio-Rad Gel Imaging System (ChemiDoc™ XRS + ; Bio-Rad Laboratories, Inc., Hercules, CA, USA) was used for densitometric analysis of the target protein bands. The Quantity One software v4.6.6 (Bio-Rad Laboratories, Inc.) was used to quantify the protein expression levels of each group.

### Statistics

SPSS software (version 22.0 IBM, Armonk, NY, USA) helped in statistical analysis. The data were expressed as means ± standard division (SD). Normality and variance homogeneity tests were assessed using SPSS. The data from two or more groups were statistically analyzed using one-way and bidirectional variance assessments, followed by post hoc adjustments using an LSD correction. MWM test and electrophysiological analysis were executed with two-way repeated measures (RM) ANOVA, followed by an LSD post hoc test. *P* < 0.05 indicated statistically significant results. The Tamhane test was used for data analyzing. Graphs were made using the software Prism (version 9.3.1).

## Results

### Voluntary exercise before or post-injury improved TBI-induced impairment in exploratory locomotor activity and anxiety-like behavior

**Fig. 1:**
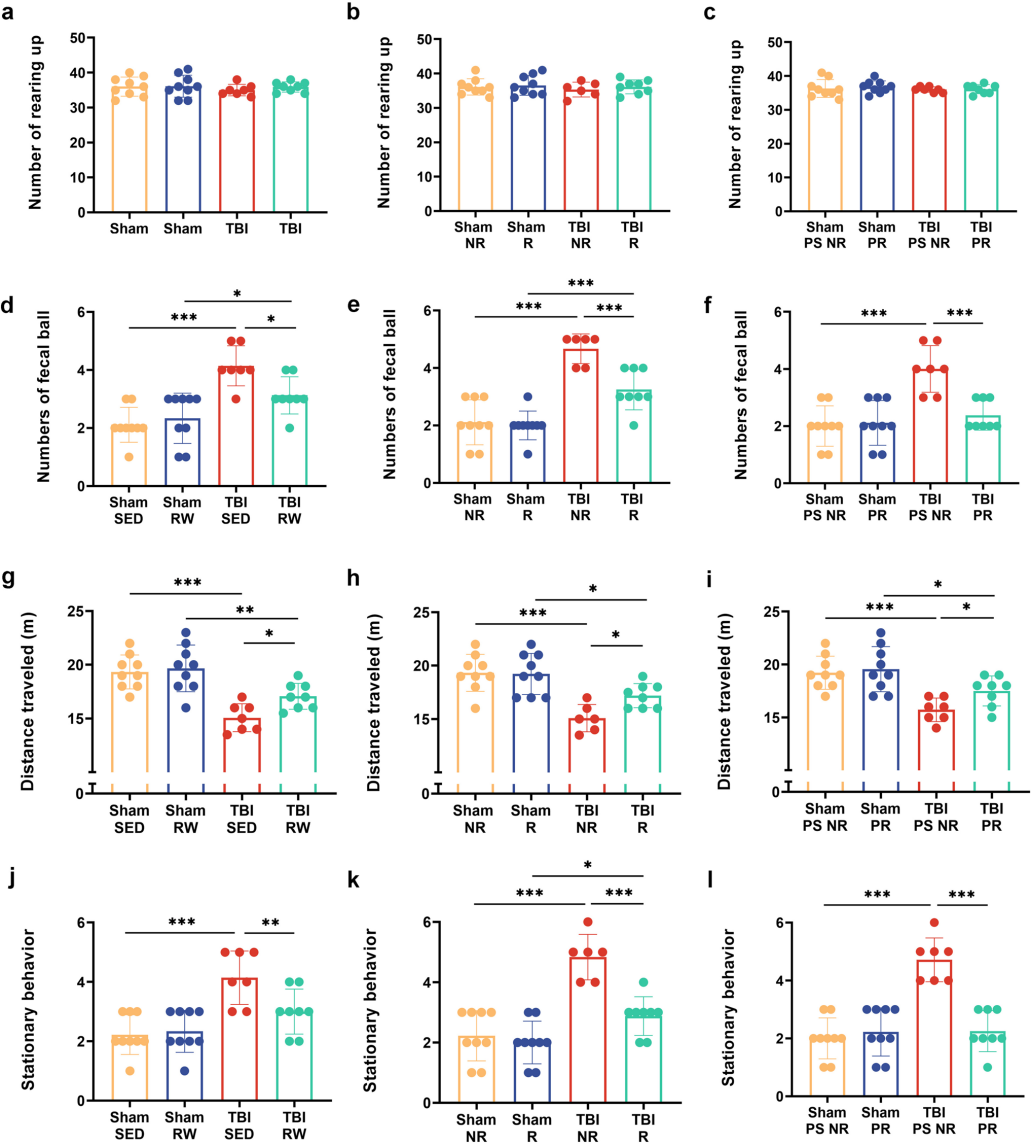
Voluntary RW exercise improved the performances of TBI mice in open field assessment. **a**–**c**, number of rearing up; **d–f**, number of fetal balls; **g**–**i**, distance traveled (**m**); (**j**–**l**), the number of stationary behaviors during the 10 min trial period. Data are expressed as Mean ± SD. Sham SED (Sham SED, mice were treated with sedentary treatment before sham surgery), Sham RW (Sham Running Wheel exercise, mice were treated with 3 weeks free running wheel exercise before sham surgery), Sham NR (Sham Non-Runner, mice were treated no running wheel training for 3 weeks after sham surgery), Sham R (Sham-Runner, mice were treated with 3 weeks of running wheel exercise training after sham surgery), Sham PS NR (Sham PreS + Non-runner, mice were treated no running wheel pre-training and post-injury training after sham surgery), Sham PR (Sham + Pre-Runner, mice were treated with 3 weeks of running wheel pre-training combined with 3 weeks of running wheel post-injury training after sham surgery); TBI SED (TBI sedentary, mice were treated with sedentary treatment before TBI surgery), TBI RW (TBI Running Wheel, mice were treated with 3 weeks free running wheel exercise before TBI surgery), TBI NR (TBI Non-Runner, mice were treated with no running wheel training for 3 weeks after TBI surgery), TBI R (TBI-Runner, mice were treated with 3 weeks of running wheel exercise training after TBI surgery), TBI PS NR (TBI PreS + Non-runner, mice were treated with no running wheel pre-training and post-injury training after TBI surgery), TBI PR (TBI + Pre-Runner, mice were treated with 3 weeks of running wheel pre-training combined with 3 weeks of running wheel post-injury training after TBI surgery)

Open field tests can depict exploratory and anxiety-like behavior and locomotor activity (Kraeuter et al. 2019). The numbers of rearing up, fecal ball, and stationary behavior during the 10-min test are measured to determine anxiety-like behavior. The total number of rearing up indicated no significant difference between the pre-exercise and age-matched SED controls groups in the Sham or TBI groups (*p* > 0.05). A similar result could be observed between the runner and non-runner controls (*p* > 0.05). No significant difference was identified between PR and PS NR controls in the Sham or TBI groups (*p* > 0.05) (Fig. 1a–c).

TBI-injured mice defecated more during tests than the mice that underwent sham operation. The mice in the TBI group (TBI RW and TBI SED, TBI NR and TBI R, TBI PS NR) showed more fecal balls than the Sham group (Sham RW and Sham SED, Sham NR and Sham R, Sham PS NR) (TBI RW vs. Sham RW,* p* < 0.05; TBI SED vs. Sham SED, *p* < 0.001; TBI NR vs. Sham NR, *p* < 0.001; TBI R vs. Sham R, *p* < 0.001; TBI PS NR vs. Sham PS NR, *p* < 0.001) (Fig. 1d–f). Moreover, the mice in the TBI RW group had fewer fecal balls than the TBI SED group (*p* < 0.05)(Fig. 1d). The TBI R group revealed a remarkably significantly declined trend with the fecal balls than the TBI NR group (*p* < 0.001) (Fig. 1e). Compared with the TBI PS NR group, the mice in the TBI PR group possessed significantly decreased fecal balls (*p* < 0.001) (Fig. 1f). In pre-training groups, the TBI SED group mice had more standing behavior than the Sham SED group (*p* < 0.001) (Fig. 1j). A significant difference could be seen in the stationary behavior between the TBI SED and TBI RW groups (*p* < 0.01) (Fig. 1j). The mice in TBI NR group mice in post-injury exercise groups showed a higher stationary behavior frequency than the Sham NR group (*p* < 0.001) (Fig. 1k). The post-injury exercise intervention decreased stationary behavior in TBI mice. The TBI R group mice had a reduced trend in stationary behavior than the TBI NR group (*p* < 0.001) (Fig. 1k). The TBI PS NR groups showed increased stationary behavior than the in Sham PS NR (*p* < 0.001) (Fig. 1l). The TBI PR group mice exhibited less stationary behavior than the TBI PS NR group (*p* < 0.001) (Fig. 1l).

These fecal ball numbers and stationary behavior results indicated that the mild TBI operation led to anxiety in mice. Moreover, the voluntary exercise implemented before and/ or after injury effectively enhanced anxiety-like behavior.

Specifically, TBI surgery significantly reduced general motor and exploratory activity in mice, however, this improved after exercise training (Fig. 1g–i). In groups that did exercise prior to operation, mice in the TBI groups (TBI SED and TBI RW) covered less compared to the sham group as measured by the total distance covered (Sham SED and Sham RW) (TBI SED vs. Sham SED, *p* < 0.001; TBI RW vs. Sham RW, *p* < 0.01) (Fig. 1g). However, the TBI SED and TBI RW exercise groups depicted nearly equal distances (*p* > 0.05) (Fig. 1g). In post-injury exercise intervention mice, the TBI group mice (TBI NR and TBI R) traveled shorter distances than the Sham NR groups (TBI NR vs. Sham NR)(TBI NR vs. Sham NR, *p* < 0.001; TBI R vs. Sham R, *p* < 0.05) (Fig. 1h). The TBI Runner groups indicated an increasing trend in total distance to the TBI Non-Runner (*p* < 0.05) (Fig. 1h). The TBI groups (TBI PS NR and TBI PR) revealed a more significant reduction in total distance than the Sham groups (Sham PS NR and Sham PR) (TBI PS NR vs. Sham PS NR, *p* < 0.001; TBI PR vs. Sham PR,* p* < 0.05) among the mixture exercise groups (Fig. 1i). TBI PR had a more elevating trend in total distance than the TBI PS NR group (*p* > 0.05) (Fig. 1i).The open-filed test indicated that the TBI operation impaired the gross locomotor and exploratory activity in mice. Before or after TBI injury, voluntary exercise showed promising effects in reversing the damage. Furthermore, TBI mice treated with voluntary exercise before and after injury significantly enhanced gross locomotor and exploratory functions.

### Simultaneous voluntary exercise before and after injury ameliorated spatial memory and recognition deficiency induced by TBI

**Fig. 2:**
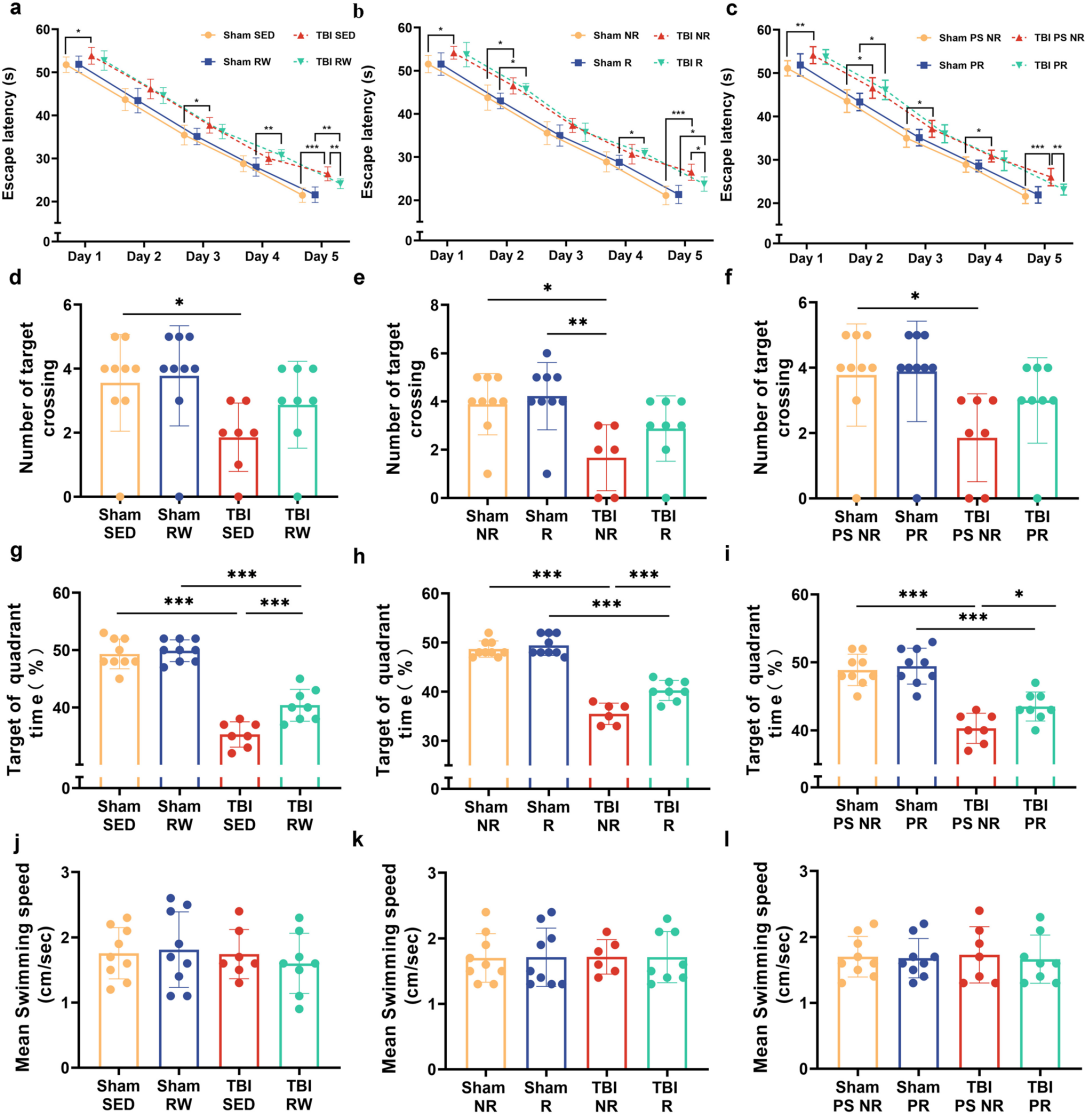
Voluntary RW exercise partially alleviated TBI-induced spatial learning and memory impairments indicated by MWM test. Two-way RM ANOVA followed by Bonferroni post hoc test were employed for the MWM test. **a–c**: the escape latency of mice in the training period from 1 to 5 days. **a**:*p < 0.05, the Sham RW group vs. the TBI RW group; ^###^*p* < 0.001, the Sham SED group vs. the TBI SED group; ^$^*p* < 0.05, the Sham SED group vs. the TBI RW group. **b**: *p < 0.05, ***p < 0.001, the Sham R group vs. the TBI NR group; ^###^*p* < 0.001, the Sham NR group vs. the TBI NR group; **c**: *p < 0.05, ***p < 0.001, the Sham PS NR group vs. the TBI PS NR group; ^###^*p* < 0.001, the Sham PR group vs. the TBI PS NR group. **d**–**f** The number of target platform crossings in the target platform during the probe test. **g**–**i** Target of quadrant time of mice in probe testing. **j**–**l** Mean swimming speed (cm/sec) of mice during probe test. Data are expressed as Mean ± SD (*n* = 9)

The MWM and NOR tests helped assess whether voluntary exercise affected spatial and recognition memory deficiencies related to TBI in mice, depicted by analysis of variance. The first five days of the hidden platform test demonstrated that the escape latency of all the groups decreased gradually with time. The TBI group mice spent more time identifying the hidden underwater platform than the age-matched mice in the Sham groups. However, RW exercise training before or after the injury can decrease the difference and significantly improve the spatial memory ability of TBI mice (TBI RW vs. TBI SED, *p* < 0.01; TBI R vs. TBI NR, *p* < 0.05)(Fig. 2a, b). The escape latency of TBI mice on the fifth day of pre-training combined with the post-injury exercise training group (TBI PR) was lower than that the pre-training combined with the sitting group (TBI PR vs. TBI PS NR, *p* < 0.01)(Fig. 2c).

On the sixth day, the platform was removed for probe testing. In TBI groups, no significant differences were observed in the number of target crossing and mean swimming speed between RW exercise training groups and without training groups (*p* > 0.05) (Fig. 2d–f, j–l). Compared with the TBI SED, TBI NR, and TBI PS NR groups, the RW training group mice had a shorter time to identify the target (TBI RW vs. TBI SED,* p* < 0.001; TBI R vs. TBI NR, *p* < 0.001; TBI PR vs. TBI PS NR,* p* < 0.05) (Fig. 2g–i).

**Fig. 3:**
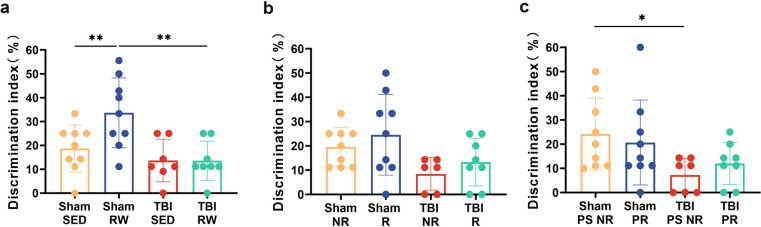
Voluntary RW exercise treated following injury restored the retrieval of object recognition memory of TBI mice. **a**–**c**: The Discrimination Index in different groups. Data are expressed as Mean ± SD (*n* = 9)

One-way RM variance analysis indicated that the exercising Sham RW mice spent more time on the NOR task than those in the Sham SED (Sham RW vs. Sham SED, *p* < 0.01)or TBI RW groups (Sham RW vs. TBI RW, *p* < 0.01) (Fig. 3a). However, no difference could be seen in the time spent on novel object exploration among the groups treated with only three weeks of exercise training and those treated with three-week pre-training and three-week exercise training after TBI operation (TBI R vs. TBI NR, *p* > 0.05; TBI PR vs. TBI PS NR, *p* > 0.05) (Fig. 3b, c).

### Simultaneous treatment with exercise before and after injury redressed the abnormal Delta and Beta Frequency Band increases due to TBI

**Fig. 4:**
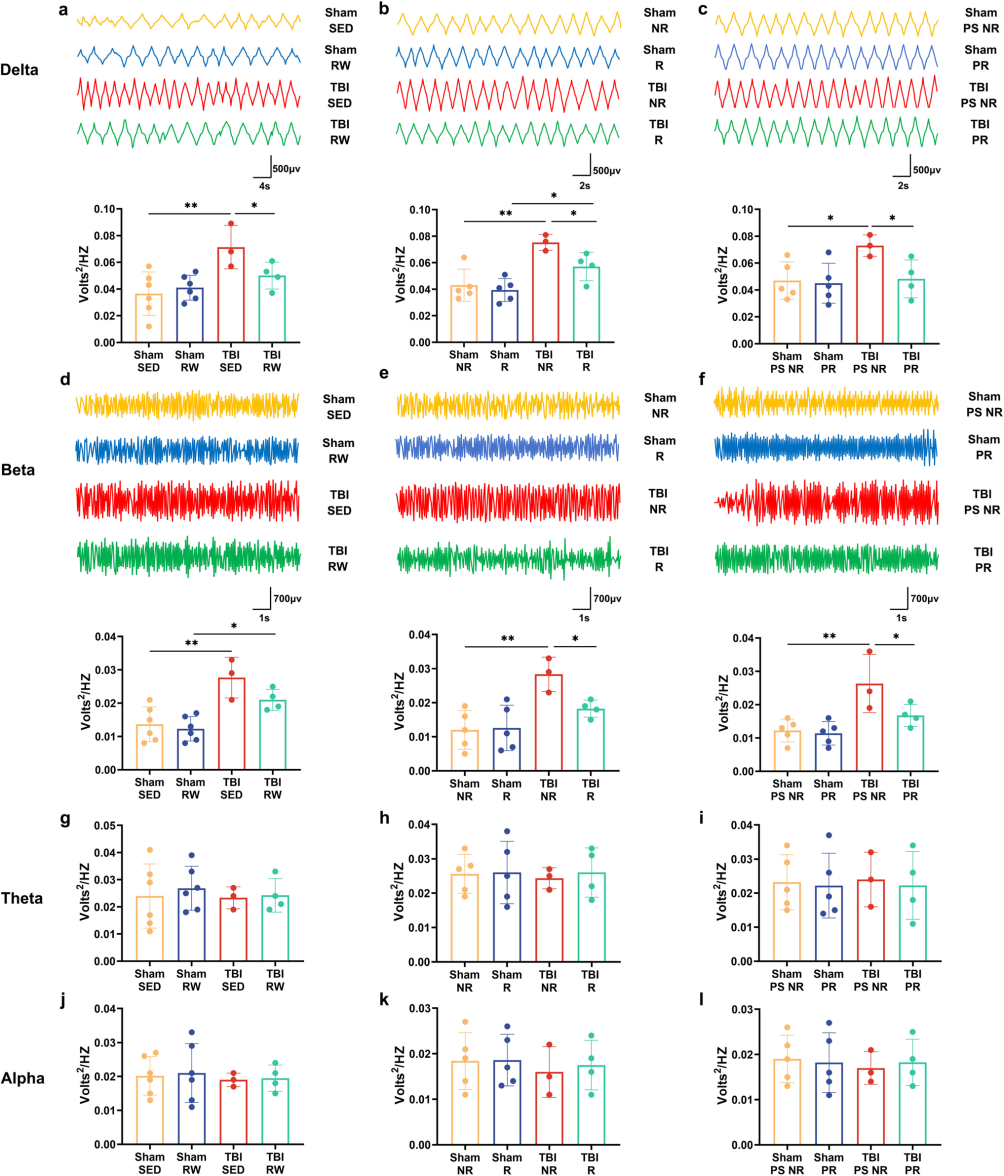
Voluntary RW exercise partially redressed the abnormal EEG recording indicated by spectral power in Sham and TBI groups on 21 dpo. **a**–**d**: delta(1–3 Hz), theta(4–7 Hz), alpha(8–14 Hz) and delta(15–35 Hz) frequency range bands during the free exercise pre-training group mice. **e**–**h**: delta theta, alpha and delta frequency range bands during the post-injury exercise intervention group mice. **i**–**l**: delta, theta, alpha and delta frequency range bands during the pre-training combined with TBI post-injury exercise training group mice. Data are expressed as Mean ± SD (*n* = 5)

The EEG recordings helped determine whether voluntary exercise impacted the functional integrity of neuronal networks among TBI mice. The results indicated that TBI SED ones had a significant increase in the delta and beta frequency band than the Sham SED group (TBI SED vs. Sham SED, *p* < 0.01) (Fig. 4a, d). The TBI RW group showed a downtrend in the delta frequency band compared with the TBI SED group (Fig. 4a, *p* < 0.05). No significant differences could be observed in theta and alpha frequency between the sedentary and RW exercise groups (Fig. 4b, c, *p* > 0.05) (Fig. 4a–d). Similar results were observed in the post-injury exercise intervention and pre-training associated with TBI post-injury exercise training groups (Fig. 4e, Sham NR vs. TBI NR, *p* < 0.01; Sham R vs. TBI R, *p* < 0.05; h, Sham NR vs. TBI NR, *p* < 0.01; i, Sham PS NR vs. TBI PS NR, *p* < 0.05; l, Sham PS NR vs. TBI PS NR, *p* < 0.01). In TBI groups, RW exercise intervention groups reduced the delta and beta frequency bands (Fig. 4a, TBI RW vs. TBI SED, *p* < 0.05; e, h, TBI R vs. TBI NR, *p* < 0.05; i, l, TBI PR vs. TBI PS NR, *p* < 0.05). However, the means are not significantly different between the TBI SED group and TBI RW group in beta frequency (*p* > 0.05) (Fig. 4d). Similarly, no evident changes could be seen in theta and alpha bands between the post-injury training intervention and the pre-training combined post-injury training intervention groups (Fig. 4f, g, and j, k, *p* > 0.05). Therefore, the TBI operation elevated the delta and beta frequency bands. In contrast, voluntary exercise treatment before and after the operation redressal those EEG parameters adequately.

### Exercise simultaneously before and after injury improved the hippocampal synaptic plasticity damage due to TBI

**Fig. 5:**
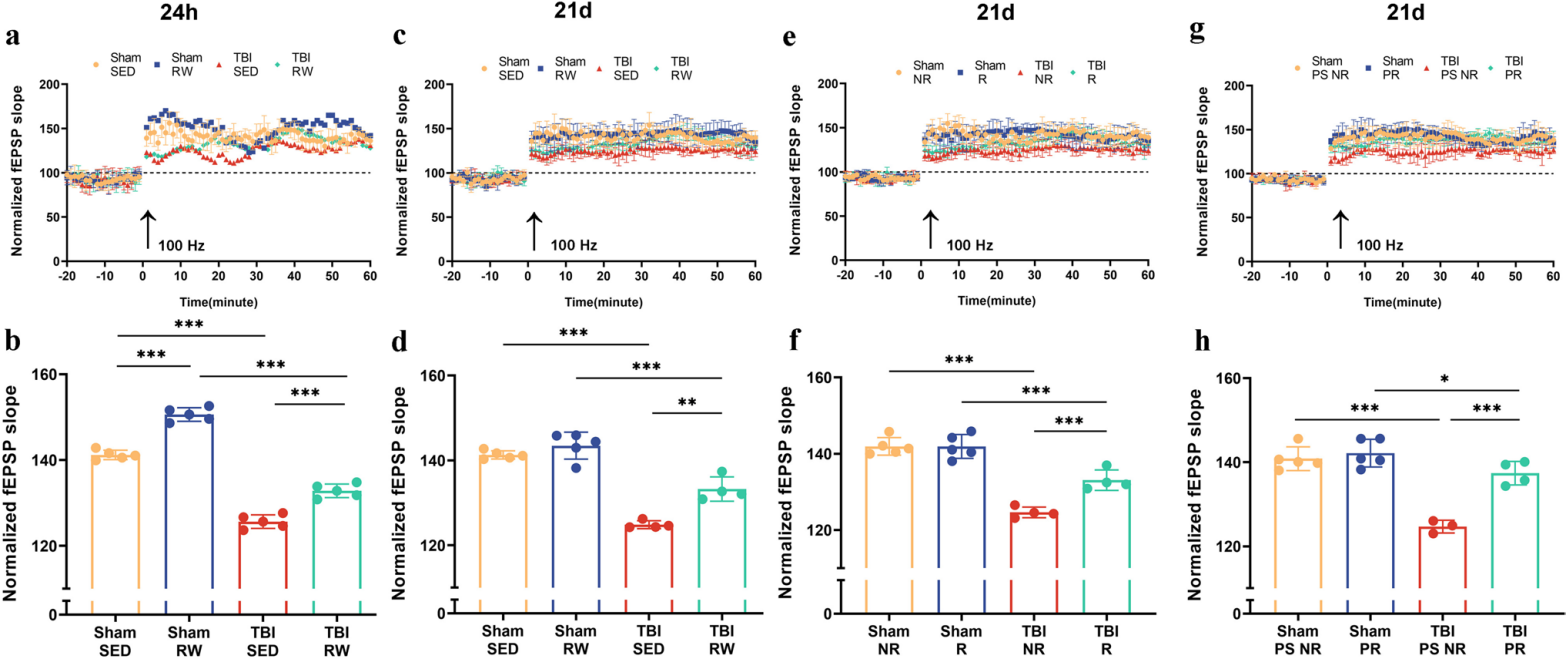
Voluntary RW exercise restored synaptic LTP of hippocampus in TBI mice. After treatment with a high-frequency, tetanic stimulation train (100 Hz, 1 s), a robust fEPSP slope was induced in mice both from Sham and TBI groups. Effect of exercise pre-training 24 h (**a**–**b**) or 21 days (**c**–**d**) after operation on LTP changes of mice. **e**–**f** Effect of post-injury exercise intervention 21 days after operation on LTP changes of mice. **g**–**h** Effect of pre-training combined with TBI post-injury exercise training 21 days after operation on LTP changes of mice. Data are expressed as Mean ± SD (*n* = 5). Two-way (RM) ANOVA followed by Bonferroni post hoc test was employed for analysis of the LTP of each group

The electrophysiology recordings validated the injury impact on the hippocampus and determined whether exercise affects the synaptic plasticity post-TBI. The results depicted that the fEPSP slope in mice was indistinguishable at 24 hpo (Fig. 5a, b) and 21 dpo (Fig. 5c–h). However, a tetanic stimulation train (100 Hz, 1 s) for 60 min post-treatment, having a high-frequency outcome and robust fEPSP slope was induced in mice. The slope magnitude varies among groups (Fig. 5b, d, f, h). The TBI mice showed a lower normalized fEPSP slope than Sham SED (TBI SED vs. Sham SED, *p* < 0.001) or Sham RW groups (TBI RW vs. Sham RW, *p* < 0.001)(Fig. 5b) in preliminary voluntary RW exercise-treated groups after 24 h of operation. In contrast, the RW exercise-treated mice possessed a higher normalized fEPSP than the sedentary groups (Fig. 5b, Sham RW vs. Sham SED, *p* < 0.001; TBI RW vs. TBI SED, *p* < 0.001). Similarly, the TBI mice indicated a decline in normalized fEPSP than in Sham SED (TBI SED vs. Sham SED, *p* < 0.001) and Sham RW groups (TBI RW vs. Sham RW, *p* < 0.001). The reason is that the mice were treated with preliminary voluntary RW exercise 21 days post-operation (Fig. 5d). In contrast, the TBI RW group had a higher fEPSP slope than the TBI SED group (Fig. 5d, *p* < 0. 01). There were lower normalized fEPSP slopes in the TBI mice than among the sham-operated mice in post-injury exercise intervention groups (Fig. 5f, TBI NR vs. Sham NR, *p* < 0.001; TBI R vs. Sham R, *p* < 0.001). In contrast, the exercise-trained TBI R mice showed a higher fEPSP slope than the TBI NR group (Fig. 5f, *p* < 0.001). The fEPSP of TBI PS NR groups was more reduced than in Sham PS NR (Fig. 5h, *p* < 0.001) or TBI PR groups (Fig. 5h, *p* < 0.001) mice who had received pre-training combined with the post-injury exercise intervention group.

Thus, TBI impaired synaptic plasticity, which was countered through exercising before or after injury. Furthermore, the synaptic plasticity was restored to the pre-injury level through simultaneous exercising simultaneously before and after the TBI operation.

### Exercise before and after injury corrected the aberrant expression increase of Nav1.1, Nav1.3, and Nav1.6 proteins due to TBI

**Fig. 6:**
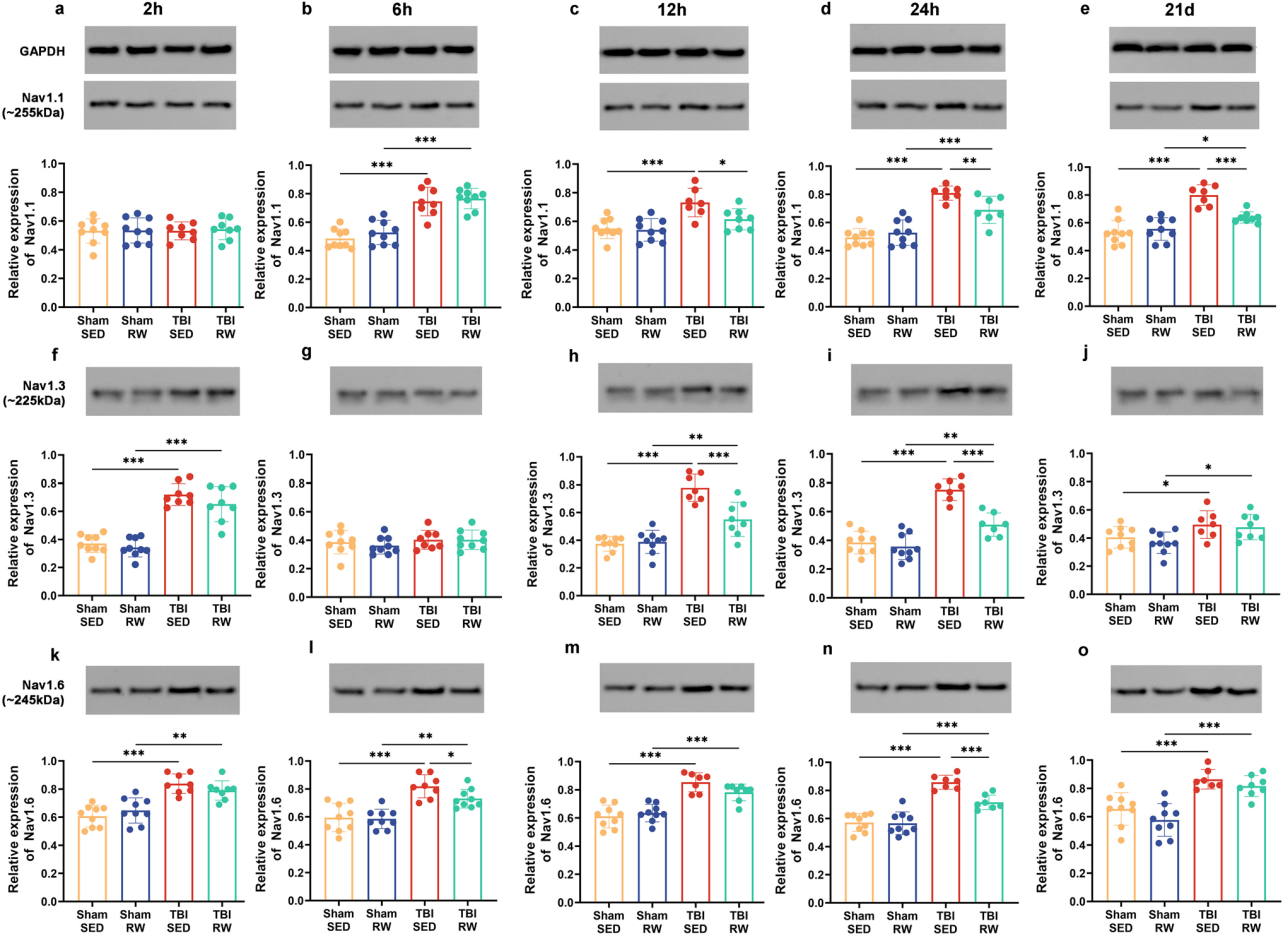
Preliminary RW exercise redressed abnormal VGSCs protein expression of TBI mice. The expression of Nav1.1 (**a**–**e**), Nav1.3 (**f**–**j**) and Nav1.6 (**k**–**o**) proteins in each group was treated with exercise pre-training at different periods, which indicated by western blotting assay. Data are expressed as Mean ± SD (*n* = 9)

In the preliminary voluntary RW group, the Nav1.1 protein expression in the hippocampus was significantly up-regulated at 6 hpo in the TBI mice than in Sham SED or RW groups (Fig. 6b, Sham SED vs. TBI SED, *p* < 0.001; Sham RW vs. TBI RW, *p* < 0.001). Nav1.1 protein expression was decreased in the TBI RW group from 12 hpo to 21 dpo when compared with the TBI SED group (Fig. 6c, *p* < 0.05; d, *p* < 0.01; e, *p* < 0.001). The TBI RW group possessed a lower Nav 1.3 expression than the TBI SED group at 12 hpo (Fig. 0.6 h, *p* < 0.001). The Nav1.3 protein expression consistently trended at 24 hpo with 12 hpo (Fig. 6h, i, *p* < 0.001). The Nav1.6 protein expression was lower in the TBI RW group than in the TBI SED group at 24 hpo (Fig. 6n, *p* < 0.001).

**Fig. 7:**
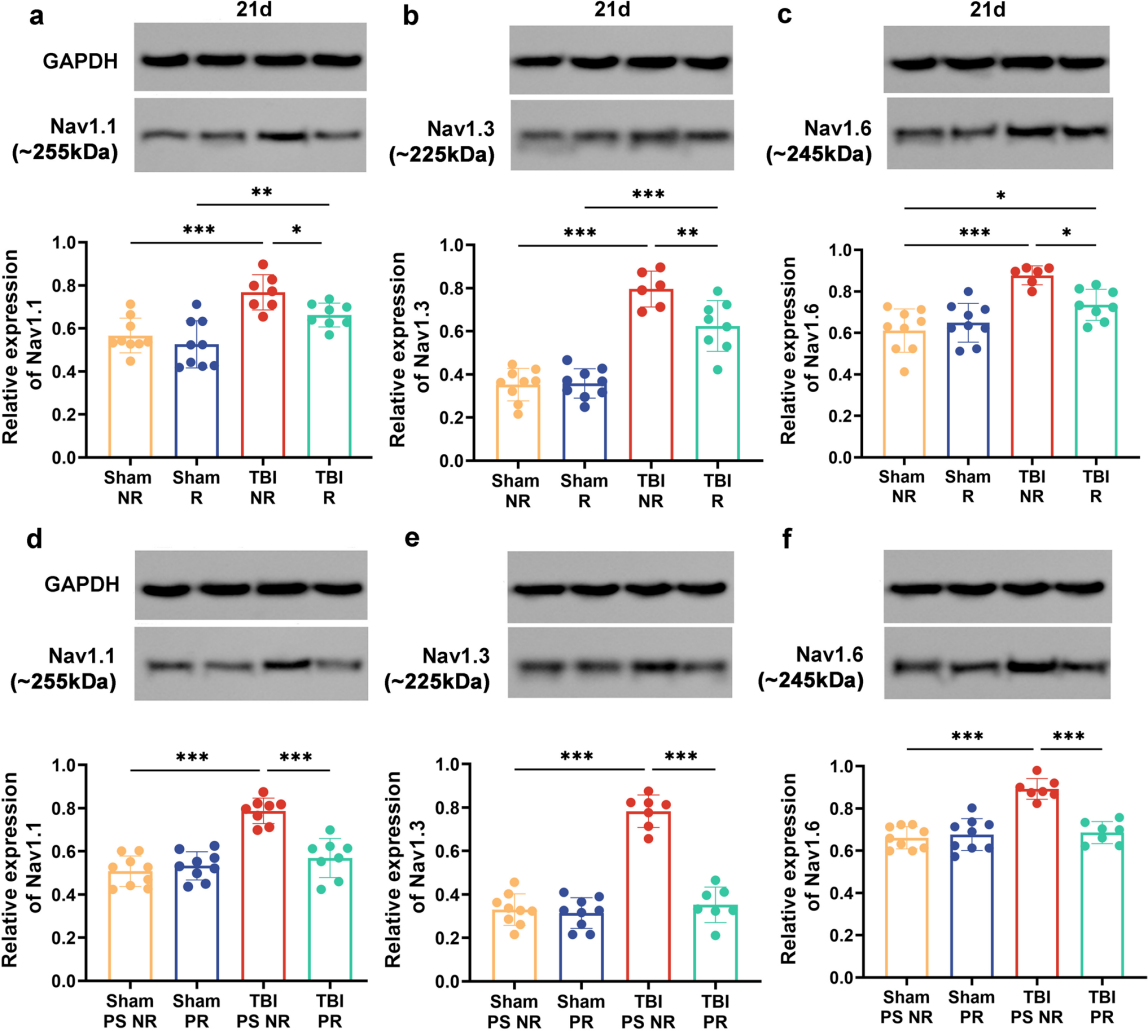
Either voluntary RW before TBI or prior-injury combined with post-injury exercise training redressed abnormal VGSCs protein expression of TBI mice. **a**–**c** The expression of Nav1.1, Nav1.3, Nav1.6 transmembrane protein in preliminary voluntary RW group. **d**–**f** The expression of Nav1.1, Nav1.3, Nav1.6 transmembrane protein in pre-training combined with TBI post-injury exercise training group. Data are expressed as Means ± SD (*n* = 9)

Nav1.1, 1.3 and 1.6 proteins expressions in the hippocampus were up-regulated within TBI NR at 21 dpo in the post-injury exercise intervention group than the Sham NR group (Fig. 7a–c, *p* < 0.001). Nav 1.1, 1.3 and 1.6 protein levels were down-regulated in the hippocampus after exercise treatment in TBI mice (Fig. 7b, *p* < 0.01; a, c, *p* < 0.05;).

Moreover, the hippocampal expression of Nav1.1, 1.3 and 1.6 proteins was significantly enhanced in TBI PS NR at 21 dpo. Simultaneously, the above indicators were considerably down-regulated in the TBI PR group (Fig. 7d–f, *p* < 0.001). Additionally, there were no significant differences in Nav1.1, 1.3, and 1.6 protein expressions between Sham PR and Sham PS NR (Fig. 7d–f).

These results indicated that TBI up-regulated Nav1.1, Nav1.3 and Nav1.6 protein expressions. In contrast, exercise treatment before and after injury reversed these changes. Furthermore, the protein expression was restored to the Sham level when exercising simultaneously before and after injury.

### The cell viability is improved in injured cortical cells cultivated inside an exercise-conditioned medium

**Fig. 8:**
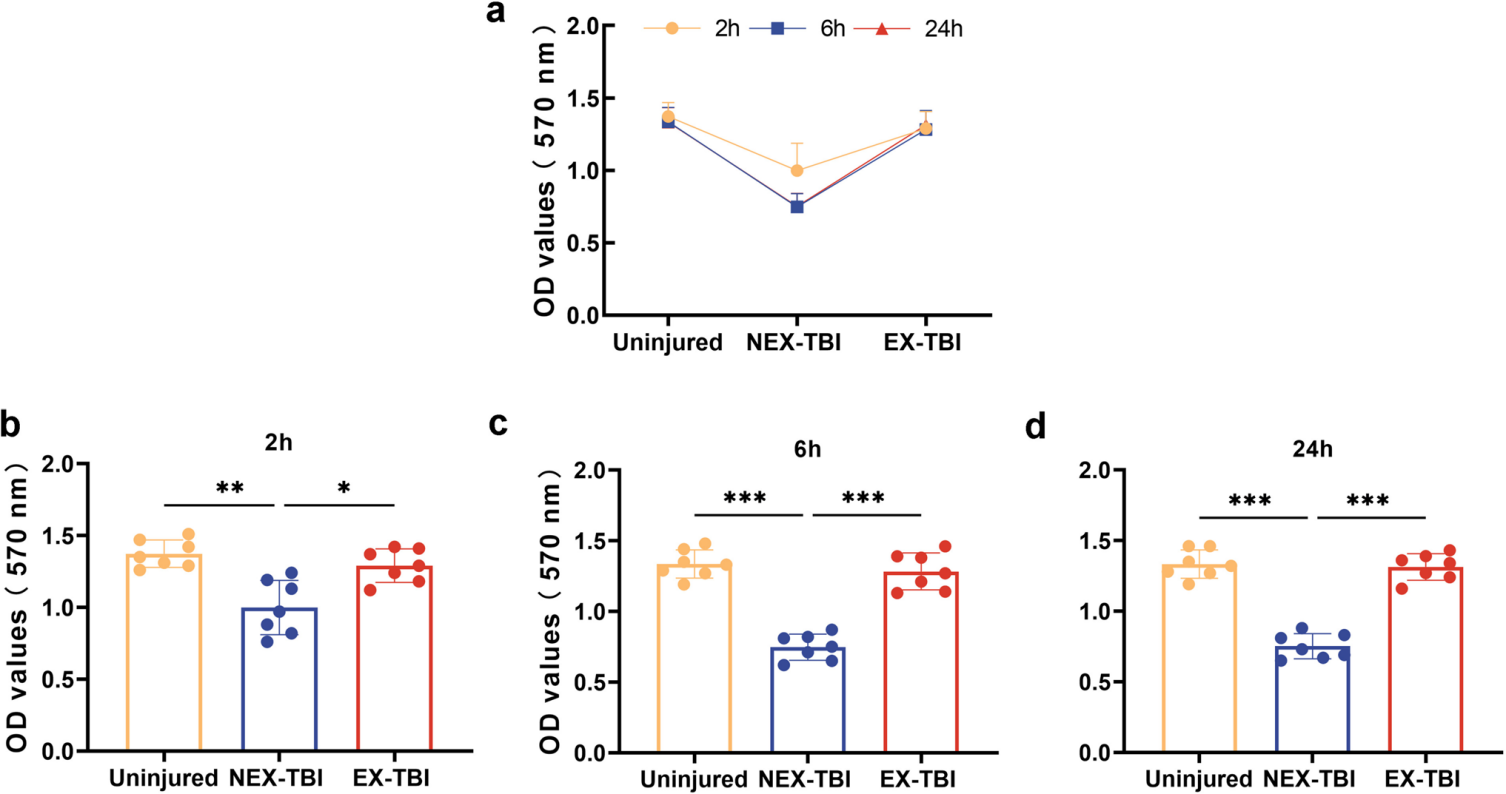
Treat with exercise-conditioned serum induced primary cortical neurons recovery following mechanical injury indicated by MTT assay. NEX-TBI: at 2, 6 or 24 h after mechanical injury, primary injured cortical neurons were treated with 1 ml of medium containing 10% non-exercise-conditioned serum instead of neurobasal medium. EX-TBI: At 2, 6 or 24 h after mechanical injury, primary injured cortical neurons were treated with 1 ml of medium containing 10% exercise-conditioned serum instead of neurobasal medium. Data are expressed as Mean ± SD (*n* = 7)

The cell viability of TBI-injured cultured cortical neurons was evaluated using the MTT assay. The injured neurons cultivated in a non-exercise-conditioned medium at 2, 6, and 24 hpo decreased cell viability compared to the uninjured ones (Fig. 8b, *p* < 0.01; c, d, *p* < 0.001). However, the cell viability of injured neurons in exercise-conditioned medium was higher than those in non-exercise-conditioned medium (Fig. 8b, *p* < 0.05; c, d, *p* < 0.001).

These results indicated that TBI impairs cortical neuron viability. Moreover, culturing cortical cells inside an exercise-conditioned medium significantly repairs the injured cortical cell viability.

### The neuronal hyperexcitability and sodium current overload caused by TBI were reversed with exercise-conditioned serum in injured cortical cell treatment

**Fig. 9:**
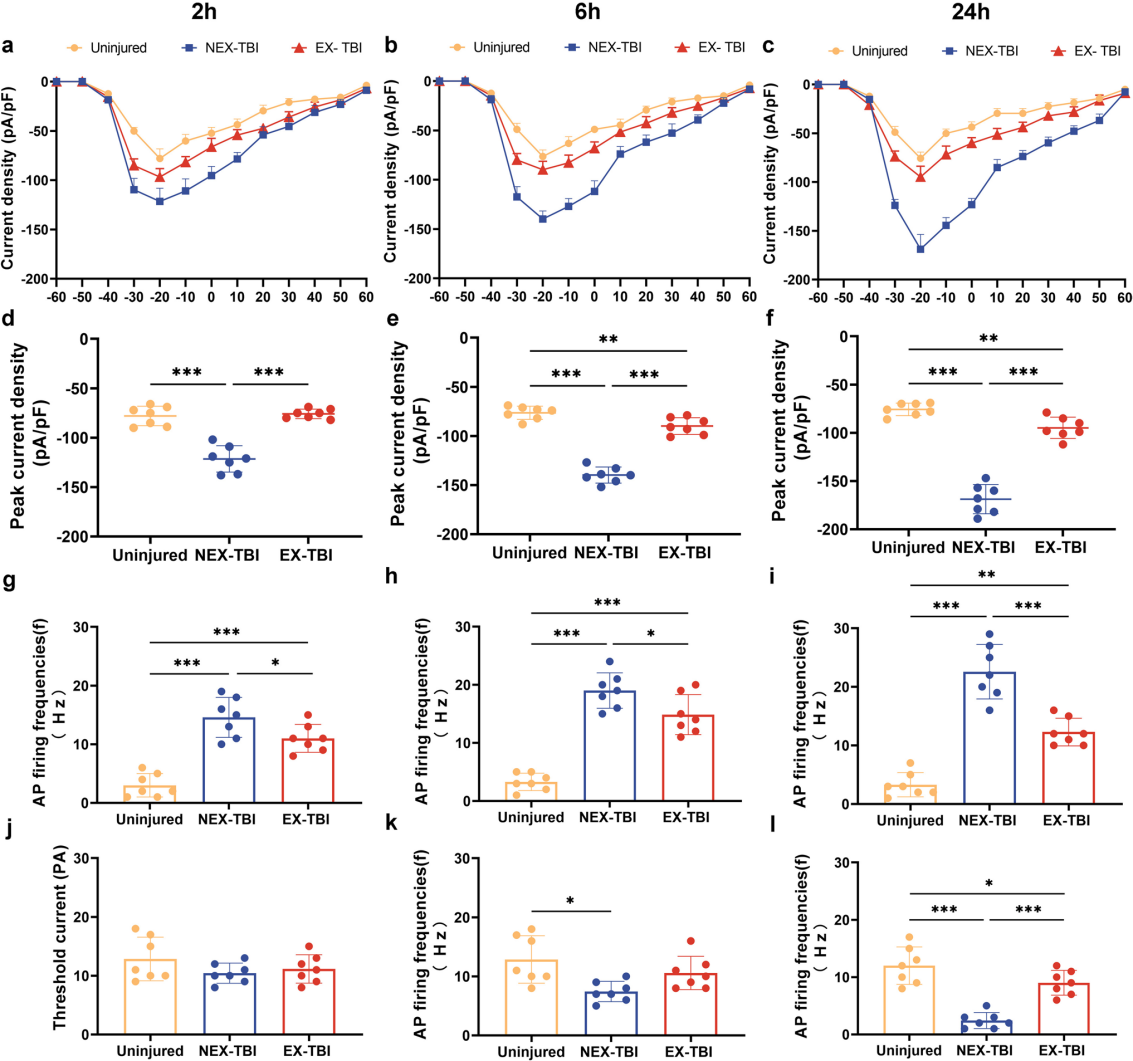
Exercise conditioned medium treatment redressed the neuronal sodium current after mechanical injury recorded using electrophysiological patch clamp. **a**–**c** Current–voltage relationships of sodium current density in cells with different treatments. **d**–**f** Peak current density. Effects of exercise training on changes in action potentials of damaged neurons provoked by TBI. **g**–**i** AP firing frequencies **j**–**l** Threshold current (PA) Data are expressed as Mean ± SD (*n* = 7)

Whole-cell patch-clamp recordings were conducted on cultured cortical cells after different treatments to identify whether exercise mice serum repressed the neuronal hyperexcitability caused by injury. The action potential frequency of impaired cortical cells inside the non-exercise-conditioned medium at 2, 6, and 24 hpo was significantly improved compared to uninjured cortical cells (Fig. 9g–i, *p* < 0.001). A significant decrease could be observed in the threshold current at 24 hpo post-injury (Fig. 9l, *p* < 0.001).The AP frequency of injured cortical cells treated using the exercise-conditioned medium at 2, 6 and 24 hpo was significantly decreased compared to those treated with the non-exercise-conditioned medium(Fig. 9g,* p* < 0.05; h, *p* < 0.05; i, *p* < 0.001). Therefore, the medium injury treatment induced neuronal hyperexcitability across injured cortical neurons, which the serum from exercise mice reduced.

Electrophysiological patch clamp recording validated whether neuronal excitability was related to the elevated sodium current. The sodium current density curves and current density in non-exercise-conditioned medium-treated ones (NEX-TBI) were significantly reduced compared to the uninjured ones at 2, 6 and 24 hpo (Fig. 9a–c). The Na^+^ and peak current densities of injured cortical cells cultivated in the exercise-conditioned medium (EX-TBI group) at 2, 6 and 24 hpo post-injury were significantly enhanced compared to those in non-exercise-conditioned medium-treated cells (NEX-TBI) (Fig. 9d–f, *p* < 0.001). Therefore, the serum from exercise mice helped restore the neuronal aberrant sodium current induced by TBI.

### Effects of exercise-conditioned serum on the aberrant status of sodium-channel proteins caused by TBI

**Fig. 10:**
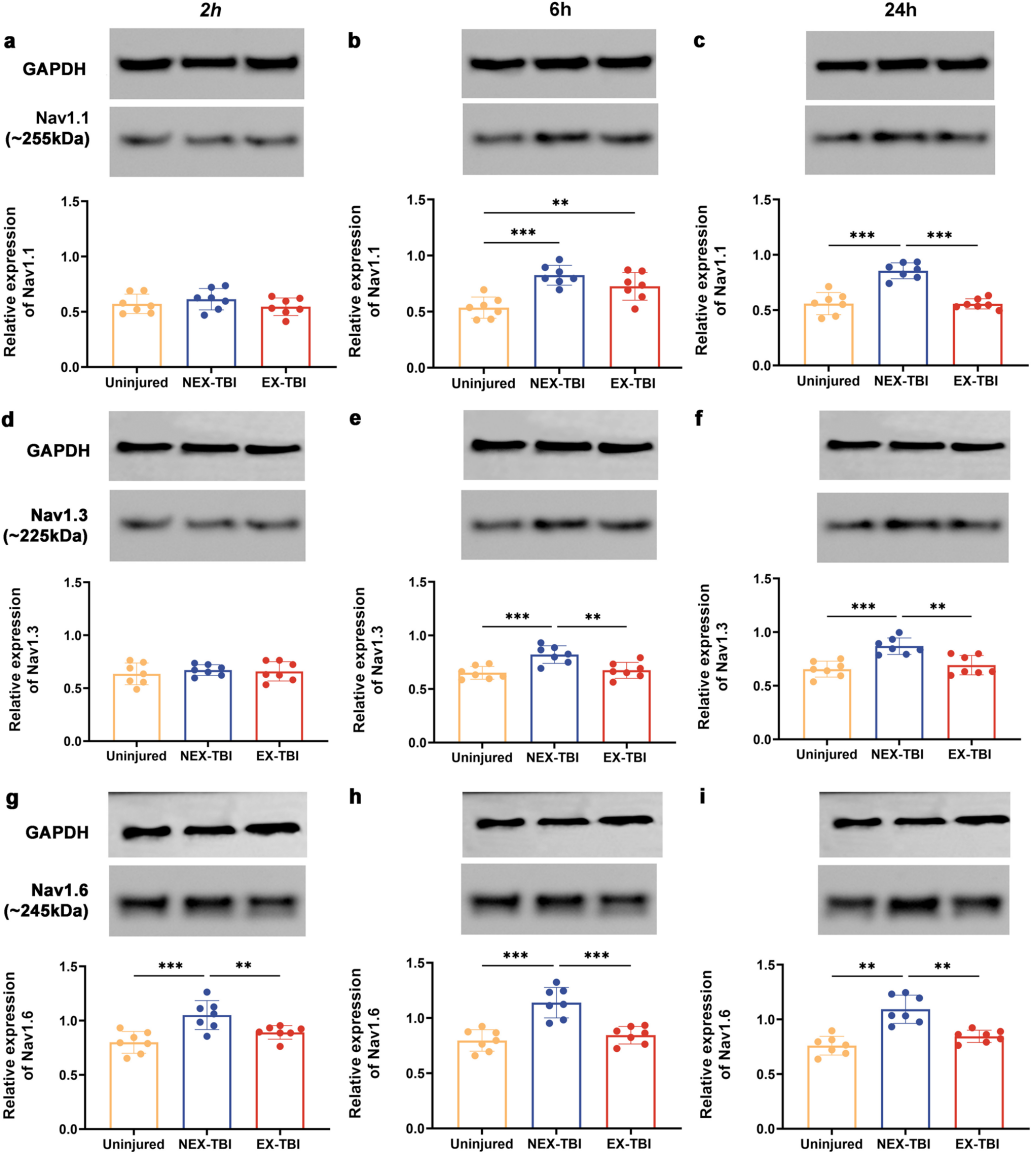
Exercise-conditioned serum treatment redressed the abnormal expressions of Nav1.1, Nav1.3, Nav1.6 proteins in cortical neurons. The Nav1.1 protein expression showed in (**a**–**c**), the Nav1.3 protein expression showed in (d-f), while the Nav1.6 protein expression showed in (**g**–**i**). Data are expressed as Mean ± SD (*n* = 7)

In uninjured and injured cultured cortical cells, the expression levels of sodium-channel proteins, such as Nav1.1, Nav1.3 and Nav1.6, were investigated to determine the underlying mechanism by which exercise mice serum redressed neuronal hyperexcitability and sodium-channel overload induced by TBI. The expression of Nav1.1, Nav1.3 and Nav1.6 in impaired cortical cells grown in the non-exercise-conditioned medium (NEX-TBI group) was significantly elevated at 6 and 24 hpo post-injury compared to those in the uninjured group (Fig. 10b, c, e, f, h, *p* < 0.001). After 24 hpo, the Nav1.1, Nav1.3, and Nav1.6 expression levels were decreased in cortical cells grown in an exercise-conditioned medium (EX-TBI group) compared to those in the non-exercise-conditioned medium (NEX-TBI group) (Fig. 10c, *p* < 0.001; f, *p* < 0.01). The differences were insignificant compared to those in the uninjured group (Fig. 10, *p* > 0.05). Moreover, the Nav1.3 expression in the EX-TBI group at 6 hpo was significantly reduced (Fig. 10e, *p* < 0.01). It was not substantially different from the uninjured cells (Fig. 10e, *p* > 0.05). In the NEX-TBI group, the Nav1.6 expression was decreased declined at 2 hpo post-injury compared to the uninjured ones (Fig. 10g, *p* < 0.001). However, Nav1.6 levels were significantly reduced after treatment with the exercise-conditioned medium at 2 hpo post-injury compared to those treated with non-exercise-conditioned medium groups (Fig. 10g, *p* < 0.01). The results from the uninjured groups showed no significant difference (Fig. 10g, *p* > 0.05).

These results depicted that the exercise-conditioned serum alleviated neuronal excitability, which could be linked with the decreasing Nav1.1, 1.3, and 1.6 expressions.

## Discussion

The present study describes the promising therapeutic effects of voluntary RW exercise in the TBI mice model and mechanically injured cortical cells in vitro. TBI mice treated with voluntary RW training before and/or after TBI improves locomotor function, emotion and cognition ability while restoring the aberrant neuronal excitability. Simultaneously, the voluntary RW exercise alters the increased VGSCs, such as Nav1.1, Nav1.3, and Nav1.6, in the ipsilateral frontal cortex and hippocampus of TBI mice. Moreover, exercise-conditioned medium treatment rescued cells through viability recovery and redressing sodium current. Moreover, up-regulated Nav1.1, Nav1.3 and Nav1.6 are resumed in injured cortical neurons after treatment with exercise-conditioned medium.

TBI is a severe global public health, with traffic accidents and accidental falls being the most common cause in younger and older adults. TBI is associated with varying consciousness degrees and cognitive impairment (Corrigan et al. 2014). Applying combinatorial biomarkers and electrophysiological techniques could highlight the brain injury mechanism, facilitating neurological function recovery. Thus, it provides a helpful reference to the rehabilitate patients.

Secondary brain injury due to the primary outcome of TBI, including ischemia and hypoxia, ion channel dysfunction, brain edema, and neuroinflammatory response, involves a complex physiological and biochemical cascade (Iaccarino et al. 2015). Previous studies have indicated that TBI can elevate the cascade reactions of different cytokines, enhance the sodium current (Na^+^) intensity and excitability of neurons, and control BDNF to suppress elevated VGSC expressions (W. Chen et al. 2017). mRNA and protein expressions of Nav1.1, Nav1.2, Nav1.3, and Nav1.6 in the nervous system of mammals were altered in the epilepsy model (Huang et al. 2013; Mao et al. 2010a, b). Therefore, sodium influx and depolarization regulated by VGSCs is an early event in a series of cellular abnormalities caused by TBI. Consequently, it is well positioned as an upstream target for pharmacological modulation of different pathological responses against TBI. Studies have established that improving neuronal plasticity and activity is essential to in the recovery post-TBI injury (Batulu et al. 2019; Trompoukis and Papatheodoropoulos 2020). Researchers have observed that physical exercise lasting more than 4 weeks could be a safe and noninvasive rehabilitation method to alleviate cognitive impairment in brain injury patients. However, the underlying mechanisms still need to be explored.

Our study depicted that exercise training treated in TBI models repaired fEPSP. Thus, it improves synaptic plasticity in the hippocampus and enhances neuronal activity, consistent with previous studies (Radahmadi et al. 2016). Thus, RW exercise training leads to recovery from TBI. The treatment improves autonomous movement and anxiety-like behaviors and enhances learning and memory.

EEG data depict that the Delta and Beta rhythms enhance after TBI. In contrast, three-week RW training treatment before and after TBI repressed abnormal EEG rhythms and restored athletic ability, spatial learning, and memory. Delta oscillations are correlated with many cognitive processes, including working memory (Harmony 2013). Elevated synchrony in the beta and delta frequency bands after TBI is associated with different alterations in deeper brain structures and dysfunctional cortical action (Atlan and Margulies 2019; Shah et al. 2017). Thus, the EEG recordings obtained in this study partially indicated the behavioral changes in TBI mice after RW exercise treatment.

TBI also reduced fEPSP slops and elevated neuronal sodium current, neuronal excitability, and Nav1.1, Nav1.3, and Nav1.6 protein expressions in the hippocampus and cortex. Similar results could be observed in primary cortical neurons cultured from the cortical and hippocampal regions of mice. RW exercise treatment for TBI redressed the abnormal EEG synchrony, Nav protein expression, and neuronal hyperexcitability. The free exercise with RW for four weeks before TBI improved the emotion-loss motor and cognitive functions of TBI mice. It suppressed neuronal loss and cortical microglia activation by inhibiting cytochrome C and Bcl-2 up-regulation and mitochondrial membrane permeability down-regulation. Thus, RW exercise training before an injury could restore neuronal activity and spatial memory ability induced by TBI in mice (Zhao et al. 2015).

Voluntary RW exercise can enhance memory ability, hippocampal neuronal excitability, and long-term potentiation (Ivy et al. 2020; Sakalem et al. 2017). Previous studies have demonstrated that the cognitive functional recovery of TBI through exercise is achieved by improving the expression of cytochrome C oxidase and BDNF in mitochondria and enhancing neuronal number and density. The cognitive improvement-related molecules and proteins remain unknown (Gu et al. 2014). Fortunately, our results identified that 3-week pre-training or post-injury training in TBI mice rectifies neuronal hyperexcitability due to Na current overload post-TBI. Moreover, improving neuronal plasticity and activity is vital in TBI injury recovery (Batulu et al. 2019; Dobryakova et al. 2020). Neuronal protections are linked with redressing neuronal hyperexcitability by VGSCs to investigate the neuronal protections produced by RW exercise, such as improvement of learning and memory. We examined the peak current density, firing frequency and threshold of AP neuronal potential in injured neurons treated with exercise-conditioned medium. Exercise-conditioned medium treatment redressed peak current density, decreased firing frequency, and enhanced the threshold current of sodium current, restoring neuronal excitability. The study demonstrates that RW exercise training enhances fEPSP slopes, elevates hippocampal synaptic plasticity, decreases AP frequency, and improves the threshold of neuronal excitability correction.

Moderate aerobic exercise inhibits TBI injury through several mechanisms. Reports depict that aerobic exercise over 2 weeks can regulate neurotrophic factor BDNF gene expressions in rats. Moreover, neuronal plasticity, spatial learning, and memory ability are also improved. Therefore, BDNF is crucial in recovering neural and cognitive function enhanced by exercise (Zafonte et al. 2018). The present study established that RW exercise training protects neuronal excitability, facilitating cognitive behavior neuroprotection. Therefore, it could be correlated with the expressional current regulation from the neuronal sodium channel proteins.

The current work indicated a novel molecular pathological training-mediated exercise mechanism to enable cognitive improvement in TBI mice, consistent with previous research (Zafonte et al. 2018). Moreover, high expression of Nav1.1α was associated with cognitive deficits in AD mouse models (Martinez-Losa et al. 2018). Nav1.3 upregulation in the damaged cortex was significantly identified 2 h post-TBI acute phase exposure in rats (Chen et al. 2014). Others revealed that inhibiting Nav1.6 channel expressions was a determinant of the Aβ_1-42_ oligomer. Moreover, there was induced membrane depolarization, elevated peak frequency, and hippocampal neuron hyperexcitability (Ciccone et al. 2019). These results indicated that blocking the Nav1.1, Nav1.3 and Nav1.6 expression partially restored cognitive dysfunction and brain disorders due to TBI. TBI mice or cortical neurons treated using voluntary RW training or exercise-conditioned medium lead to improved action function, learning and memory ability, neuronal activity recovery, or neuronal activity regulation. The abnormal increase in Nav1.1, Nav1.3, and Nav1.6 levels corrected the neuronal excitability and Na current. Therefore, exercise training for TBI mice could be a promising strategy for TBI treatment and counter the cognitive deficits and neuronal hyperexcitation.

### Supplementary Information

Below is the link to the electronic supplementary material.

Supplementary file1 (DOCX 50 KB)

Supplementary file2 (DOCX 24 KB)

## Data Availability

The data used to support the findings of this study are available from the corresponding author upon reasonable request.

## References

[CR1] Atlan LS, Margulies SS (2019). Frequency-dependent changes in resting state electroencephalogram functional networks after traumatic brain injury in piglets. J Neurotrauma.

[CR2] Bao TH, Miao W, Han JH, Yin M, Yan Y, Wang WW, Zhu YH (2014). Spontaneous running wheel improves cognitive functions of mouse associated with miRNA expressional alteration in hippocampus following traumatic brain injury. J Mol Neurosci.

[CR3] Batulu H, Du GJ, Li DZ, Sailike D, Fan YH, Geng D (2019). Effect of poly-arginine R18 on neurocyte cell growth via autophagy in traumatic brain injury. Exp Ther Med.

[CR4] Chen D, Yang Z, Xia C, Huang Y, Yin B, Guo F, Huang J (2014). The effect of Lactobacillus rhamnosus hsryfm 1301 on the intestinal microbiota of a hyperlipidemic rat model. BMC Complement Altern Med.

[CR5] Chen W, Sheng J, Guo J, Peng G, Hong J, Li B, Wang S (2017). Cytokine cascades induced by mechanical trauma injury alter voltage-gated sodium channel activity in intact cortical neurons. J Neuroinflamm.

[CR6] Chytrova G, Ying Z, Gomez-Pinilla F (2008). Exercise normalizes levels of MAG and Nogo-A growth inhibitors after brain trauma. Eur J Neurosci.

[CR7] Ciccone R, Franco C, Piccialli I, Boscia F, Casamassa A, de Rosa V, Pannaccione A (2019). Amyloid β-induced upregulation of na(v)1.6 underlies neuronal hyperactivity in Tg2576 Alzheimer’s disease mouse model. Sci Rep.

[CR8] Corrigan JD, Cuthbert JP, Harrison-Felix C, Whiteneck GG, Bell JM, Miller AC, Pretz CR (2014). US population estimates of health and social outcomes 5 years after rehabilitation for traumatic brain injury. J Head Trauma Rehabil.

[CR9] Dobryakova YV, Stepanichev MY, Markevich VA, Bolshakov AP (2020). Long-term potentiation in the hippocampal CA3 to CA1 synapses may be induced in vivo by activation of septal cholinergic inputs. Int J Neurosci.

[CR10] Figueiredo CP, Clarke JR, Ledo JH, Ribeiro FC, Costa CV, Melo HM, Ferreira ST (2013). Memantine rescues transient cognitive impairment caused by high-molecular-weight Aβ oligomers but not the persistent impairment induced by low-molecular-weight oligomers. J Neurosci.

[CR11] Fox GB, Fan L, Levasseur RA, Faden AI (1998). sustained sensory/motor and cognitive deficits with neuronal apoptosis following controlled cortical impact brain injury in the mouse. J Neurotrauma.

[CR12] Gao G, Wu X, Feng J, Hui J, Mao Q, Lecky F, Jiang J (2020). Clinical characteristics and outcomes in patients with traumatic brain injury in China: a prospective, multicentre, longitudinal, observational study. Lancet Neurol.

[CR13] Gu YL, Zhang LW, Ma N, Ye LL, de Wang X, Gao X (2014). Cognitive improvement of mice induced by exercise prior to traumatic brain injury is associated with cytochrome c oxidase. Neurosci Lett.

[CR14] Harmony T (2013). The functional significance of delta oscillations in cognitive processing. Front Integr Neurosci.

[CR15] Hazra A, Macolino C, Elliott MB, Chin J (2014). Delayed thalamic astrocytosis and disrupted sleep-wake patterns in a preclinical model of traumatic brain injury. J Neurosci Res.

[CR16] Hojman P, Dethlefsen C, Brandt C, Hansen J, Pedersen L, Pedersen BK (2011). Exercise-induced muscle-derived cytokines inhibit mammary cancer cell growth. Am J Physiol Endocrinol Metab.

[CR17] Hu T, Zhou F, Chang Y, Li Y, Liu G, Hong Y, Bao T (2015). miR21 is associated with the cognitive improvement following voluntary running wheel exercise in TBI mice. J Mol Neurosci.

[CR18] Hu T, Xiao Z, Mao R, Chen B, Lu M, Tong J, Xiyang Y (2017). Navβ2 knockdown improves cognition in APP/PS1 mice by partially inhibiting seizures and APP amyloid processing. Oncotarget.

[CR19] Hu T, Li S, Liang WQ, Li SS, Lu MN, Chen B, Wang XY (2020). Notoginsenoside R1-induced neuronal repair in models of alzheimer disease is associated with an alteration in neuronal hyperexcitability, which is regulated by Nav. Front Cell Neurosci.

[CR20] Huang XJ, Mao Q, Lin Y, Feng JF, Jiang JY (2013). Expression of voltage-gated sodium channel Nav1.3 is associated with severity of traumatic brain injury in adult rats. J Neurotrauma.

[CR21] Iaccarino MA, Bhatnagar S, Zafonte R (2015). Rehabilitation after traumatic brain injury. Handb Clin Neurol.

[CR22] Ivy AS, Yu T, Kramár E, Parievsky S, Sohn F, Vu T (2020). A unique mouse model of early life exercise enables hippocampal memory and synaptic plasticity. Sci Rep.

[CR23] Iwata A, Stys PK, Wolf JA, Chen X-H, Taylor AG, Meaney DF, Smith DH (2004). Traumatic axonal injury induces proteolytic cleavage of the voltage-gated sodium channels modulated by tetrodotoxin and protease inhibitors. J Neurosci.

[CR24] Katano H, Fujita K, Kato T, Asai K, Kawamura Y, Masago A, Yamada K (1999). Traumatic injury in vitro induces IEG mRNAs in cultured glial cells, suppressed by co-culture with neurons. NeuroReport.

[CR25] Khellaf A, Khan DZ, Helmy A (2019). Recent advances in traumatic brain injury. J Neurol.

[CR26] Kim HJ, Magrané J (2011). Isolation and culture of neurons and astrocytes from the mouse brain cortex. Methods Mol Biol.

[CR27] Kraeuter A-K, Guest PC, Sarnyai ZN (2019). The open field test for measuring locomotor activity and anxiety-like behavior. Methods Mol Biol.

[CR28] Lee M, Kim D, Shin H, Sung H, Choi JH (2011). High-density EEG recordings of the freely moving mice using polyimide-based microelectrode. J Vis Exp.

[CR29] Lee SY, Amatya B, Judson R, Truesdale M, Reinhardt JD, Uddin T, Khan F (2019). Clinical practice guidelines for rehabilitation in traumatic brain injury: a critical appraisal. Brain Inj.

[CR30] Lourenco MV, Clarke JR, Frozza RL, Bomfim TR, Forny-Germano L, Batista AF, Felice FGD (2013). TNF-α mediates PKR-dependent memory impairment and brain IRS-1 inhibition induced by Alzheimer’s β-amyloid oligomers in mice and monkeys. Cell Metab.

[CR31] Manzanares G, Brito-da-Silva G, Gandra PG (2018). Voluntary wheel running: patterns and physiological effects in mice. Braz J Med Biol Res.

[CR32] Manzanares G, Brito-da-Silva G, Gandra PG (2020). The voltage-gated sodium channel inhibitor, 4,9-anhydrotetrodotoxin, blocks human Na v 1.1 in addition to Na v 1.6. Neurosci Lett.

[CR33] Mao Q, Jia F, Zhang X-H, Qiu Y-M, Ge J-W, Bao W-J, Jiang J-Y (2010). The up-regulation of voltage-gated sodium channel Nav1.6 expression following fluid percussion traumatic brain injury in rats. Neurosurgery.

[CR34] Mao Q, Jia F, Zhang XH, Qiu YM, Ge JW, Bao WJ, Jiang JY (2010) The up-regulation of voltage-gated sodium channel Nav1.6 expression following fluid percussion traumatic brain injury in rats. Neurosurgery, 66(6), 1134–1139; discussion 1139. 10.1227/01.Neu.0000369612.31946.A2 10.1227/01.NEU.0000369612.31946.A2 20421839

[CR35] Martinez-Losa M, Tracy TE, Ma K, Verret L, Clemente-Perez A, Khan AS, Palop JJ (2018). Nav1.1-Overexpressing interneuron transplants restore brain rhythms and cognition in a mouse model of Alzheimer’s disease. Neuron.

[CR36] Mori T, Wang X, Jung J-C, Sumii T, Singhal AB, Fini E, Lo EH (2002). Mitogen-activated protein kinase inhibition in traumatic brain injury: in vitro and in vivo effects. J Cereb Blood Flow Metab.

[CR37] Radahmadi M, Hosseini N, Alaei H (2016). Effect of exercise, exercise withdrawal, and continued regular exercise on excitability and long-term potentiation in the dentate gyrus of hippocampus. Brain Res.

[CR38] Sakalem ME, Seidenbecher T, Zhang M, Saffari R, Kravchenko M, Wördemann S, Ambrée O (2017). Environmental enrichment and physical exercise revert behavioral and electrophysiological impairments caused by reduced adult neurogenesis. Hippocampus.

[CR39] Shah SA, Mohamadpour M, Askin G, Nakase-Richardson R, Stokic DS, Sherer M, Schiff ND (2017). Focal electroencephalographic changes index post-traumatic confusion and outcome. J Neurotrauma.

[CR40] Shan S, Wang S, Song X, Khashaveh A, Lu Z, Dhiloo KH, Zhang Y (2019). Antennal ionotropic receptors IR64a1 and IR64a2 of the parasitoid wasp Microplitis mediator (Hymenoptera: Braconidate) collaboratively perceive habitat and host cues. Insect Biochem Mol Biol.

[CR41] Szu JI, Chaturvedi S, Patel DD, Binder DK (2020). Aquaporin-4 dysregulation in a controlled cortical impact injury model of posttraumatic epilepsy. Neuroscience.

[CR42] Szu JI, Patel DD, Chaturvedi S, Lovelace JW, Binder DK (2020). Modulation of posttraumatic epileptogenesis in aquaporin-4 knockout mice. Epilepsia.

[CR43] Tan Y, Liu G, Chen H, Lu M, Chen B, Hu T, Xiyang Y (2020). Exercise-induced cognitive improvement is associated with sodium channel-mediated excitability in APP/PS1 mice. Neural Plast.

[CR44] Trompoukis G, Papatheodoropoulos C (2020). Dorsal-ventral differences in modulation of synaptic transmission in the hippocampus. Front Synaptic Neurosci.

[CR45] Wolf JA, Stys PK, Lusardi T, Meaney D, Smith DH (2001). Traumatic axonal injury induces calcium influx modulated by tetrodotoxin-sensitive sodium channels. J Neurosci.

[CR46] XiYang Y, Wang Y, Zhao Y, Ru J, Lu B, Zhang Y, Wang T (2016). Sodium channel voltage-gated beta 2 plays a vital role in brain aging associated with synaptic plasticity and expression of COX5A and FGF-2. Mol Neurobiol.

[CR47] Zafonte RD, Shih SL, Iaccarino MA, Tan CO (2018). Neurologic benefits of sports and exercise. Handb Clin Neurol.

[CR48] Zhao Z, Sabirzhanov B, Wu J, Faden AI, Stoica BA (2015). Voluntary exercise preconditioning activates multiple antiapoptotic mechanisms and improves neurological recovery after experimental traumatic brain injury. J Neurotrauma.

