# Supplementary material for: Voluntary running wheel exercise induces cognitive improvement post traumatic brain injury in mouse model through redressing aberrant excitation regulated by voltage-gated sodium channels 1.1, 1.3, and 1.6
Source: Exp Brain Res. 2023 Nov 23;242(1):205–24. doi: 10.1007/s00221-023-06734-2 (PMC10786980; doi:10.1007/s00221-023-06734-2)
Supplement: Supplementary file 1 — Supplementary file1 (DOCX 50 KB) [file 221_2023_6734_MOESM1_ESM.docx]

**Table 1. Descriptives of EEG recordings in each group ^a^**

|  | Group | N | Mean ± SD | *F* value | *P* value |
| --- | --- | --- | --- | --- | --- |
| **Delta** | **3 weeks pre-exercise training group** |  |  | 5.193 | 0.012 |
|  | Sham SED | 6 | 0.037 ± 0.016 |  |  |
|  | Sham RW | 6 | 0.041 ± 0.009 |  |  |
|  | TBI SED | 3 | 0.071 ± 0.016 |  |  |
|  | TBI RW | 4 | 0.050 ± 0.010 |  |  |
|  | **3 weeks after TBI exercise training group** |  |  | 9.652 | 0.001 |
|  | Sham NR | 5 | 0.043 ± 0.012 |  |  |
|  | Sham R | 5 | 0.039 ± 0.009 |  |  |
|  | TBI NR | 3 | 0.075 ± 0.006 |  |  |
|  | TBI R | 4 | 0.057 ± 0.011 |  |  |
|  | **3 weeks pre-training +3 weeks post-injury training** |  |  | 3.166 | 0.061 |
|  | Sham PS NR | 5 | 0.047 ± 0.014 |  |  |
|  | Sham PR | 5 | 0.045 ± 0.015 |  |  |
|  | TBI PS NR | 3 | 0.073 ± 0.008 |  |  |
|  | TBI PR | 4 | 0.048 ± 0.014 |  |  |
| **Beta** | **3 weeks pre-exercise training group** |  |  | 9.805 | 0.001 |
|  | Sham SED | 6 | 0.014 ± 0.005 |  |  |
|  | Sham RW | 6 | 0.012 ± 0.003 |  |  |
|  | TBI SED | 3 | 0.028 ± 0.006 |  |  |
|  | TBI RW | 4 | 0.021 ± 0.003 |  |  |
|  | **3 weeks after TBI exercise training group** |  |  | 7.014 | 0.005 |
|  | Sham NR | 5 | 0.012 ± 0.006 |  |  |
|  | Sham R | 5 | 0.013 ± 0.007 |  |  |
|  | TBI NR | 3 | 0.028 ± 0.005 |  |  |
|  | TBI R | 4 | 0.018 ± 0.003 |  |  |
|  | **3 weeks pre-training +3 weeks post-injury training** |  |  | 7.650 | 0.003 |
|  | Sham PS NR | 5 | 0.012 ± 0.003 |  |  |
|  | Sham PR | 5 | 0.011 ± 0.004 |  |  |
|  | TBI PS NR | 3 | 0.026 ± 0.009 |  |  |
|  | TBI PR | 4 | 0.017 ± 0.003 |  |  |
| **Theta** | **3 weeks pre-exercise training group** |  |  | 0.155 | 0.925 |
|  | Sham SED | 6 | 0.024 ± 0.012 |  |  |
|  | Sham RW | 6 | 0.027 ± 0.008 |  |  |
|  | TBI SED | 3 | 0.023 ± 0.004 |  |  |
|  | TBI RW | 4 | 0.024 ± 0.006 |  |  |
|  | **3 weeks after TBI exercise training group** |  |  | 0.043 | 0.988 |
|  | Sham NR | 5 | 0.026 ± 0.006 |  |  |
|  | Sham R | 5 | 0.026 ± 0.009 |  |  |
|  | TBI NR | 3 | 0.024 ± 0.003 |  |  |
|  | TBI R | 4 | 0.026 ± 0.007 |  |  |
|  | **3 weeks pre-training +3 weeks post-injury training** |  |  | 0.034 | 0.991 |
|  | Sham PS NR | 5 | 0.023 ± 0.008 |  |  |
|  | Sham PR | 5 | 0.022 ± 0.010 |  |  |
|  | TBI PS NR | 3 | 0.024 ± 0.008 |  |  |
|  | TBI PR | 4 | 0.022 ± 0.010 |  |  |
| **Alpha** | **3 weeks pre-exercise training group** |  |  | 0.084 | 0.968 |
|  | Sham SED | 6 | 0.020 ± 0.006 |  |  |
|  | Sham RW | 6 | 0.021 ± 0.009 |  |  |
|  | TBI SED | 3 | 0.019 ± 0.002 |  |  |
|  | TBI RW | 4 | 0.020 ± 0.004 |  |  |
|  | **3 weeks after TBI exercise training group** |  |  | 0.150 | 0.928 |
|  | Sham NR | 5 | 0.018 ± 0.006 |  |  |
|  | Sham R | 5 | 0.019 ± 0.006 |  |  |
|  | TBI NR | 3 | 0.016 ± 0.006 |  |  |
|  | TBI R | 4 | 0.018 ± 0.005 |  |  |
|  | **3 weeks pre-training +3 weeks post-injury training** |  |  | 0.084 | 0.968 |
|  | Sham PS NR | 5 | 0.019 ± 0.005 |  |  |
|  | Sham PR | 5 | 0.018 ± 0.007 |  |  |
|  | TBI PS NR | 3 | 0.017 ± 0.004 |  |  |
|  | TBI PR | 4 | 0.018 ± 0.005 |  |  |

1. **The data of *F* value and *P* value were statistically analyzed using one-way ANOVA.**

**Table 2. Test of Homogeneity of Variances of EEG recordings in each group ^a^**

|  | Group |  | *P* value |
| --- | --- | --- | --- |
| **Delta** | **3 weeks pre-exercise training group**  (Sham SED, Sham RW, TBI SED, TBI RW) | Based on Mean | 0.419 |
|  |  | Based on Median | 0.530 |
|  |  | Based on Median and with adjusted df | 0.536 |
|  |  | Based on trimmed mean | 0.425 |
|  | **3 weeks after TBI exercise training group**  (Sham NR, Sham R, TBI NR, TBI R) | Based on Mean | 0.804 |
|  |  | Based on Median | 0.939 |
|  |  | Based on Median and with adjusted df | 0.939 |
|  |  | Based on trimmed mean | 0.856 |
|  | **3 weeks after TBI exercise training group**  (Sham PS NR, Sham PR, TBI PS NR, TBI PR) | Based on Mean | 0.611 |
|  |  | Based on Median | 0.824 |
|  |  | Based on Median and with adjusted df | 0.824 |
|  |  | Based on trimmed mean | 0.628 |
| **Beta** | **3 weeks pre-exercise training group**  (Sham SED, Sham RW, TBI SED, TBI RW) | Based on Mean | 0.383 |
|  |  | Based on Median | 0.582 |
|  |  | Based on Median and with adjusted df | 0.591 |
|  |  | Based on trimmed mean | 0.393 |
|  | **3 weeks after TBI exercise training group**  (Sham NR, Sham R, TBI NR, TBI R) | Based on Mean | 0.186 |
|  |  | Based on Median | 0.380 |
|  |  | Based on Median and with adjusted df | 0.388 |
|  |  | Based on trimmed mean | 0.195 |
|  | **3 weeks after TBI exercise training group**  (Sham PS NR, Sham PR, TBI PS NR, TBI PR) | Based on Mean | 0.106 |
|  |  | Based on Median | 0.460 |
|  |  | Based on Median and with adjusted df | 0.493 |
|  |  | Based on trimmed mean | 0.118 |
| **Theta** | **3 weeks pre-exercise training group**  (Sham SED, Sham RW, TBI SED, TBI RW) | Based on Mean | 0.082 |
|  |  | Based on Median | 0.097 |
|  |  | Based on Median and with adjusted df | 0.100 |
|  |  | Based on trimmed mean | 0.083 |
|  | **3 weeks after TBI exercise training group**  (Sham NR, Sham R, TBI NR, TBI R) | Based on Mean | 0.162 |
|  |  | Based on Median | 0.264 |
|  |  | Based on Median and with adjusted df | 0.281 |
|  |  | Based on trimmed mean | 0.168 |
|  | **3 weeks after TBI exercise training group**  (Sham PS NR, Sham PR, TBI PS NR, TBI PR) | Based on Mean | 0.873 |
|  |  | Based on Median | 0.942 |
|  |  | Based on Median and with adjusted df | 0.941 |
|  |  | Based on trimmed mean | 0.882 |
| **Alpha** | **3 weeks pre-exercise training group**  (Sham SED, Sham RW, TBI SED, TBI RW) | Based on Mean | 0.145 |
|  |  | Based on Median | 0.156 |
|  |  | Based on Median and with adjusted df | 0.182 |
|  |  | Based on trimmed mean | 0.145 |
|  | **3 weeks after TBI exercise training group**  (Sham NR, Sham R, TBI NR, TBI R) | Based on Mean | 0.963 |
|  |  | Based on Median | 0.980 |
|  |  | Based on Median and with adjusted df | 0.980 |
|  |  | Based on trimmed mean | 0.963 |
|  | **3 weeks after TBI exercise training group**  (Sham PS NR, Sham PR, TBI PS NR, TBI PR) | Based on Mean | 0.512 |
|  |  | Based on Median | 0.746 |
|  |  | Based on Median and with adjusted df | 0.746 |
|  |  | Based on trimmed mean | 0.518 |

1. **The data of *P* value were statistically analyzed using bidirectional variance assessments.**

**Table 3. Multiple Comparisons of EEG recordings in each group ^a^**

|  | Group | Group | *P* value |
| --- | --- | --- | --- |
| **Delta** | **3 weeks pre-exercise training group** | |  |
|  | Sham SED | Sham RW | 0.561 |
|  |  | TBI SED | 0.002 |
|  |  | TBI RW | 0.132 |
|  | Sham RW | Sham SED | 0.561 |
|  |  | TBI SED | 0.005 |
|  |  | TBI RW | 0.305 |
|  | TBI SED | Sham SED | 0.002 |
|  |  | Sham RW | 0.005 |
|  |  | TBI RW | 0.050 |
|  | TBI RW | Sham SED | 0.132 |
|  |  | Sham RW | 0.305 |
|  |  | TBI SED | 0.050 |
|  | **3 weeks after TBI exercise training group** | |  |
|  | Sham NR | Sham R | 0.580 |
|  |  | TBI NR | 0.001 |
|  |  | TBI R | 0.058 |
|  | Sham R | Sham NR | 0.580 |
|  |  | TBI NR | 0.000 |
|  |  | TBI R | 0.021 |
|  | TBI NR | Sham NR | 0.001 |
|  |  | Sham R | 0.000 |
|  |  | TBI R | 0.032 |
|  | TBI R | Sham NR | 0.058 |
|  |  | Sham R | 0.021 |
|  |  | TBI NR | 0.032 |
|  | **3 weeks pre-training +3 weeks post-injury training** | |  |
|  | Sham PS NR | Sham PR | 0.819 |
|  |  | TBI PS NR | 0.021 |
|  |  | TBI PR | 0.893 |
|  | Sham PR | Sham PS NR | 0.819 |
|  |  | TBI PS NR | 0.014 |
|  |  | TBI NR | 0.726 |
|  | TBI PS NR | Sham PS NR | 0.021 |
|  |  | Sham PR | 0.014 |
|  |  | TBI PR | 0.032 |
|  | TBI PR | Sham PS NR | 0.893 |
|  |  | Sham PR | 0.726 |
|  |  | TBI PS NR | 0.032 |
| **Beta** | **3 weeks pre-exercise training group** | |  |
|  | Sham SED | Sham RW | 0.617 |
|  |  | TBI SED | 0.001 |
|  |  | TBI RW | 0.024 |
|  | Sham RW | Sham SED | 0.617 |
|  |  | TBI SED | 0.000 |
|  |  | TBI RW | 0.010 |
|  | TBI SED | Sham SED | 0.001 |
|  |  | Sham RW | 0.000 |
|  |  | TBI RW | 0.073 |
|  | TBI RW | Sham SED | 0.024 |
|  |  | Sham RW | 0.010 |
|  |  | TBI SED | 0.073 |
|  | **3 weeks after TBI exercise training group** | |  |
|  | Sham NR | Sham R | 0.863 |
|  |  | TBI NR | 0.001 |
|  |  | TBI R | 0.107 |
|  | Sham R | Sham NR | 0.863 |
|  |  | TBI NR | 0.002 |
|  |  | TBI R | 0.142 |
|  | TBI NR | Sham NR | 0.001 |
|  |  | Sham R | 0.002 |
|  |  | TBI R | 0.029 |
|  | TBI R | Sham NR | 0.107 |
|  |  | Sham R | 0.142 |
|  |  | TBI NR | 0.029 |
|  | **3 weeks pre-training +3 weeks post-injury training** | |  |
|  | Sham PS NR | Sham PR | 0.790 |
|  |  | TBI PS NR | 0.001 |
|  |  | TBI PR | 0.169 |
|  | Sham PR | Sham PS NR | 0.790 |
|  |  | TBI PS NR | 0.001 |
|  |  | TBI NR | 0.110 |
|  | TBI PS NR | Sham PS NR | 0.001 |
|  |  | Sham PR | 0.001 |
|  |  | TBI PR | 0.018 |
|  | TBI PR | Sham PS NR | 0.169 |
|  |  | Sham PR | 0.110 |
|  |  | TBI PS NR | 0.018 |
| **Theta** | **3 weeks pre-exercise training group** | |  |
|  | Sham SED | Sham RW | 0.587 |
|  |  | TBI SED | 0.916 |
|  |  | TBI RW | 0.966 |
|  | Sham RW | Sham SED | 0.587 |
|  |  | TBI SED | 0.584 |
|  |  | TBI RW | 0.657 |
|  | TBI SED | Sham SED | 0.916 |
|  |  | Sham RW | 0.584 |
|  |  | TBI RW | 0.894 |
|  | TBI RW | Sham SED | 0.966 |
|  |  | Sham RW | 0.657 |
|  |  | TBI SED | 0.894 |
|  | **3 weeks after TBI exercise training group** | |  |
|  | Sham NR | Sham R | 0.929 |
|  |  | TBI NR | 0.807 |
|  |  | TBI R | 0.933 |
|  | Sham R | Sham NR | 0.929 |
|  |  | TBI NR | 0.748 |
|  |  | TBI R | 1.000 |
|  | TBI NR | Sham NR | 0.807 |
|  |  | Sham R | 0.748 |
|  |  | TBI R | 0.759 |
|  | TBI R | Sham NR | 0.933 |
|  |  | Sham R | 1.000 |
|  |  | TBI NR | 0.759 |
|  | **3 weeks pre-training +3 weeks post-injury training** | |  |
|  | Sham PS NR | Sham PR | 0.863 |
|  |  | TBI PS NR | 0.905 |
|  |  | TBI PR | 0.877 |
|  | Sham PR | Sham PS NR | 0.863 |
|  |  | TBI PS NR | 0.788 |
|  |  | TBI NR | 0.994 |
|  | TBI PS NR | Sham PS NR | 0.905 |
|  |  | Sham PR | 0.788 |
|  |  | TBI PR | 0.803 |
|  | TBI PR | Sham PS NR | 0.877 |
|  |  | Sham PR | 0.994 |
|  |  | TBI PS NR | 0.803 |
| **Alpha** | **3 weeks pre-exercise training group** | |  |
|  | Sham SED | Sham RW | 0.821 |
|  |  | TBI SED | 0.796 |
|  |  | TBI RW | 0.871 |
|  | Sham RW | Sham SED | 0.821 |
|  |  | TBI SED | 0.658 |
|  |  | TBI RW | 0.716 |
|  | TBI SED | Sham SED | 0.796 |
|  |  | Sham RW | 0.658 |
|  |  | TBI RW | 0.918 |
|  | TBI RW | Sham SED | 0.871 |
|  |  | Sham RW | 0.716 |
|  |  | TBI SED | 0.918 |
|  | **3 weeks after TBI exercise training group** | |  |
|  | Sham NR | Sham R | 0.957 |
|  |  | TBI NR | 0.580 |
|  |  | TBI R | 0.820 |
|  | Sham R | Sham NR | 0.957 |
|  |  | TBI NR | 0.549 |
|  |  | TBI R | 0.781 |
|  | TBI NR | Sham NR | 0.580 |
|  |  | Sham R | 0.549 |
|  |  | TBI R | 0.740 |
|  | TBI R | Sham NR | 0.820 |
|  |  | Sham R | 0.781 |
|  |  | TBI NR | 0.740 |
|  | **3 weeks pre-training +3 weeks post-injury training** | |  |
|  | Sham PS NR | Sham PR | 0.821 |
|  |  | TBI PS NR | 0.625 |
|  |  | TBI PR | 0.841 |
|  | Sham PR | Sham PS NR | 0.821 |
|  |  | TBI PS NR | 0.769 |
|  |  | TBI NR | 0.989 |
|  | TBI PS NR | Sham PS NR | 0.625 |
|  |  | Sham PR | 0.769 |
|  |  | TBI PR | 0.770 |
|  | TBI PR | Sham PS NR | 0.841 |
|  |  | Sham PR | 0.989 |
|  |  | TBI PS NR | 0.770 |

1. **The data of *P* value between groups were statistically analyzed by post hoc adjustments using an with a LSD correction.**
