# Supplementary material for: Voluntary running wheel exercise induces cognitive improvement post traumatic brain injury in mouse model through redressing aberrant excitation regulated by voltage-gated sodium channels 1.1, 1.3, and 1.6
Source: Exp Brain Res. 2023 Nov 23;242(1):205–24. doi: 10.1007/s00221-023-06734-2 (PMC10786980; doi:10.1007/s00221-023-06734-2)
Supplement: Supplementary file 2 — Supplementary file2 (DOCX 24 KB) [file 221_2023_6734_MOESM2_ESM.docx]

**Journal name： Experimental Brain Research**

**Article title： Voluntary running wheel exercise induces cognitive improvement post traumatic brain injury in mouse model through redressing aberrant excitation regulated by Voltage-gated sodium channels 1.1, 1.3 and 1.6**

Dan Wang^1+^, Hui-Xiang Zhang^1+^, Guo-Ji Yan^1^, Hao-Ran Zhao^1^, Xiao-Han Dong^1^, Ya-Xin Tan^1,3^, Shan Li^1,2^, Min-Nan Lu^4^, Rong Mei^5^, Li-Na Liu^1^, Xu-Yang Wang^6*^, Yan-Bin Xiyang^1*^

^1^Institute of Neuroscience, Faculty of Basic Medical Science, Kunming Medical University, Kunming, 650500, Yunnan, China

^2^Department of Anatomy, Changsha Medical University, Changsha, China^.^

^3^Department of Pediatrics, The People's Liberation Army (PLA) Rocket Force Characteristic Medical Center, Beijing, 100088, China

^4^Science and Technology Achievement Incubation Center, Kunming Medical University, Kunming, Yunnan, 650500, China

^5^Department of Neurology, The First People’s Hospital of Yunnan Province, Kunming, Yunnan, China

^6^Department of Neurosurgery, Shanghai Sixth People's Hospital Affiliated to Shanghai Jiao Tong University School of Medicine, Shanghai, 200233, P.R. China

^+^ These authors contributed equally to this work.

Corresponding Author: Prof. Xu-Yang Wang, Department of Neurosurgery, Shanghai Sixth People's Hospital Affiliated to Shanghai Jiao Tong University School of Medicine, Shanghai, 200233, P.R. China.

E-mail addresses: [wangxuyanglxl@163.com](mailto:wangxuyanglxl@163.com)

Corresponding Author: Prof. Yan-Bin Xiyang, Institute of Neuroscience, Kunming Medical University; 1168 West Chunrong Road, Yuhua Avenue, Chenggong, Kunming, Yunnan, 650500, P.R. China

E-mail addresses: [xiyangyanbin@kmmu.edu.cn](mailto:xiyangyanbin@kmmu.edu.cn)

**Table 1 Grouping of TBI mice treated with preliminary voluntary running wheel (RW) exercise for various lab and experimental procedures**

| **Lab/behavioral**  **investigations**    **Grouping of mice** | | | **Behavior evaluation (OF/NOR/MWM)** | **EEG recording** | **LTP recording** | **Navs expression analysis**  **(Western blotting)** |
| --- | --- | --- | --- | --- | --- | --- |
|  |  |  | **Number of mice** | | | |
| **sham** | **Sedentary control** | **2 hpo** | **-** | **-** | **-** | **9** |
|  |  | **6 hpo** | **-** | **-** | **-** | **9** |
|  |  | **12 hpo** | **-** | **-** | **-** | **9** |
|  |  | **24 hpo** | **-** | **-** | **5** | **9** |
|  |  | **21 dpo** | **9** | **5** | **5** | **9** |
|  | **RW exercise** | **2 hpo** | **-** | **-** | **-** | **9** |
|  |  | **6 hpo** | **-** | **-** | **-** | **9** |
|  |  | **12 hpo** | **-** | **-** | **-** | **9** |
|  |  | **24 hpo** | **-** | **-** | **5** | **9** |
|  |  | **21 dpo** | **9** | **5** | **5** | **9** |
| **TBI** | **Sedentary control** | **2 hpo** | **-** | **-** | **-** | **8*** |
|  |  | **6 hpo** | **-** | **-** | **-** | **8*** |
|  |  | **12 hpo** | **-** | **-** | **-** | **7*** |
|  |  | **24 hpo** | **-** | **-** | **5** | **7*** |
|  |  | **21 dpo** | **7*** | **5** | **5** | **7*** |
|  | **RW exercise** | **2 hpo** | **-** | **-** | **-** | **8*** |
|  |  | **6 hpo** | **-** | **-** | **-** | **9** |
|  |  | **12 hpo** | **-** | **-** | **-** | **8*** |
|  |  | **24 hpo** | **-** | **-** | **5** | **7*** |
|  |  | **21 dpo** | **8*** | **5** | **5** | **8*** |

hours post operation (hpo), days post operation (dpo)

Mice were housed with voluntary access to a running wheel (RW, RW exercise) or an immobilized RW (Sedentary control) for 3 weeks before injury.

* Mice that died in this group were dropped out. The number of mice shows the actual number included in the analysis at the end of the experimental procedures.

OF, open field; NOR, Novel object recognition task; MWM, Morris water maze test; EEG, electroencephalogram; LTP, long-term potential.

**Table 2 Grouping of mice treated with voluntary running wheel (RW) exercise for 3 weeks post TBI for various lab and experimental procedures**

| **Lab/behavioral**  **investigations**    **Grouping of mice** | | **Behavior evaluation (OF/NOR/MWM)** | **EEG recording** | **LTP recording** | **Navs expression analysis**  **(Western blotting)** |
| --- | --- | --- | --- | --- | --- |
|  |  | **Number of mice** | | | |
| **sham** | **Non-runner** | **9** | **5** | **5** | **9** |
|  | **Runner** | **9** | **5** | **5** | **9** |
| **TBI** | **Non-runner** | **6*** | **5** | **5** | **6*** |
|  | **Runner** | **8*** | **5** | **5** | **8*** |

* Mice that died in this group were dropped out. The number of mice shows the actual number included in the analysis at the end of the experimental procedures.

TBI or sham-operated mice were housed with voluntary access to a running wheel (RW, runner) or an immobilized RW ( Non-runner) for 3 weeks.

OF, open field; NOR, Novel object recognition task; MWM, Morris water maze test; EEG, electroencephalogram; LTP, long-term potential.

**Table 3 Grouping of mice treated with voluntary running wheel (RW) exercise before TBI for 3 weeks and post TBI for 3 weeks for various lab and experimental procedures**

| **Lab/behavioral**  **investigations**    **Grouping of mice** | | **Behavior evaluation (OF/NOR/MWM)** | **EEG recording** | **LTP recording** | **Navs expression analysis**  **(Western blotting)** |
| --- | --- | --- | --- | --- | --- |
|  |  | **Number of mice** | | | |
| **Sham** | **PreS+Non-runner** | **9** | **5** | **5** | **9** |
|  | **Pre+Runner** | **9** | **5** | **5** | **9** |
| **TBI** | **PreS+Non-runner** | **7*** | **5** | **5** | **7*** |
|  | **Pre+Runner** | **8*** | **5** | **5** | **7*** |

* Mice that died in this group were dropped out. The number of mice shows the actual number included in the analysis at the end of the experimental procedures.

TBI or sham-operated mice were housed with voluntary access to a running wheel (RW) or an immobilized RW before operation for 3 weeks and post operation for 3 weeks.

OF, open field; NOR, Novel object recognition task; MWM, Morris water maze test; EEG, electroencephalogram; LTP, long-term potential.

Pre, preliminary voluntary running wheel (RW) exercise

PreS, preliminary Sedentary control
